# Investigation of LKB1 Ser^431^ phosphorylation and Cys^433^ farnesylation using mouse knockin analysis reveals an unexpected role of prenylation in regulating AMPK activity

**DOI:** 10.1042/BJ20131324

**Published:** 2014-01-20

**Authors:** Vanessa P. Houde, Maria Stella Ritorto, Robert Gourlay, Joby Varghese, Paul Davies, Natalia Shpiro, Kei Sakamoto, Dario R. Alessi

**Affiliations:** *MRC Protein Phosphorylation and Ubiquitylation Unit, College of Life Sciences, University of Dundee, Dow Street, Dundee DD1 5EH, U.K.

**Keywords:** 5-amino-4-imidazolecarboxamide riboside (AICAR), AMP-activated protein kinase-related kinase (AMPK-related kinase), monoclonal antibody, phenformin, SAD-A/SAD-B kinase, signal transduction, ACC, acetyl-CoA carboxylase, ACTH, adrenocorticotropic hormone, AICAR, 5-amino-4-imidazolecarboxamide riboside, AMPK, AMP-activated protein kinase, ARK5, AMPK-related protein kinase 5, BDNF, brain-derived neurotrophic factor, BiP, immunoglobulin heavy-chain-binding protein, BRSK, BR serine/threonine kinase, DMEM, Dulbecco's modified Eagle's medium, EDL, extensor digitorum longus, ER, endoplasmic reticulum, ERK, extracellular-signal-regulated kinase, GAPDH, glyceraldehyde-3-phosphate dehydrogenase, HA, haemagglutinin, HEK, human embryonic kidney, HRP, horseradish peroxidase, HSP90, heat-shock protein 90, MAPK, mitogen-activated protein kinase, MAPKAPK, MAPKAP (MAPK-activated protein) kinase, MARK4, MAP/microtubule affinity-regulating kinase 4, MEF, mouse embryonic fibroblast, mTOR, mammalian target of rapamycin, NUAK1, NUAK family, SNF1-like kinase, PDK1, phosphoinositide-dependent kinase 1, PH, pleckstrin homology, PI3K, phosphoinositide 3-kinase, PKA, cAMP-dependent protein kinase, raptor, regulatory associated protein of mTOR, RSK, ribosomal S6 kinase, SIK3, salt-inducible kinase 3, STRAD, STE20-related kinase adaptor, TBC1D1, TBC (Tre-2/Bub2/Cdc16) domain family, member 1

## Abstract

The LKB1 tumour suppressor protein kinase functions to activate two isoforms of AMPK (AMP-activated protein kinase) and 12 members of the AMPK-related family of protein kinases. The highly conserved C-terminal residues of LKB1 are phosphorylated (Ser^431^) by PKA (cAMP-dependent protein kinase) and RSK (ribosomal S6 kinase) and farnesylated (Cys^433^) within a CAAX motif. To better define the role that these post-translational modifications play, we created homozygous LKB1^S431A/S431A^ and LKB1^C433S/C433S^ knockin mice. These animals were viable, fertile and displayed no overt phenotypes. Employing a farnesylation-specific monoclonal antibody that we generated, we established by immunoprecipitation that the vast majority, if not all, of the endogenous LKB1 is prenylated. Levels of LKB1 localized at the membrane of the liver of LKB1^C433S/C433S^ mice and their fibroblasts were reduced substantially compared with the wild-type mice, confirming that farnesylation plays a role in mediating membrane association. Although AMPK was activated normally in the LKB1^S431A/S431A^ animals, we unexpectedly observed in all of the examined tissues and cells taken from LKB1^C433S/C433S^ mice that the basal, as well as that induced by the AMP-mimetic AICAR (5-amino-4-imidazolecarboxamide riboside), AMPK activation, phenformin and muscle contraction were significantly blunted. This resulted in a reduced ability of AICAR to inhibit lipid synthesis in primary hepatocytes isolated from LKB1^C433S/C433S^ mice. The activity of several of the AMPK-related kinases analysed [BRSK1 (BR serine/threonine kinase 1), BRSK2, NUAK1 (NUAK family, SNF1-like kinase 1), SIK3 (salt-inducible kinase 3) and MARK4 (MAP/microtubule affinity-regulating kinase 4)] was not affected in tissues derived from LKB1^S431A/S431A^ or LKB1^C433S/C433S^ mice. Our observations reveal for the first time that farnesylation of LKB1 is required for the activation of AMPK. Previous reports have indicated that a pool of AMPK is localized at the plasma membrane as a result of myristoylation of its regulatory AMPKβ subunit. This raises the possibility that LKB1 farnesylation and myristoylation of AMPKβ might promote the interaction and co-localization of these enzymes on a two-dimensional membrane surface and thereby promote efficient activation of AMPK.

## Abstract 2

Our observations reveal a significant role for the farnesylation of LKB1 being required for the efficient activation of AMPK. Our findings also cast doubt on previous claims that phosphorylation of Ser^431^ was essential for orchestrating neuronal cell polarity and embryo development.

## INTRODUCTION

Inactivating mutations in the *LKB1* kinase tumour suppressor gene cause the inherited Peutz–Jeghers cancer syndrome, in which patients are predisposed to developing benign and malignant tumours [1]. Loss-of-function mutations in LKB1 are also observed in certain sporadic cancers [2] especially lung adenocarcinomas [3,4]. LKB1 is activated through its ability to form a heterotrimeric complex with the pseudokinase STRAD (STE20-related kinase adaptor) and the scaffolding protein MO25 [5–8].

Most data have suggested that LKB1 exerts its physiological effects by phosphorylating and activating a group of 14 related protein kinases that belong to the AMPK (AMP-activated protein kinase) subfamily [9]. These include the two isoforms of the AMPK catalytic subunit (AMPKα1 and AMPKα2), which are activated following phosphorylation of their T-loop residue (Thr^172^) by LKB1 [10–12]. One of the key physiological/pathological conditions that leads to the activation of AMPK is low energy, where increasing levels of AMP and/or ADP interact with the CBS motifs of the regulatory AMPKγ subunit of AMPK [13,14]. This induces conformational changes that directly stimulate AMPKα catalytic activity through allosteric mechanisms and also promote the phosphorylation of Thr^172^ by inhibiting dephosphorylation of this residue by protein phosphatases [15,16]. Once activated, AMPKα1 and AMPKα2 function to restore and maintain energy levels by phosphorylating a myriad of proteins that control processes including cell growth and proliferation and metabolism [17].

The 12 other kinases activated by LKB1 are collectively termed the AMPK-related kinases [18]. LKB1 also activates the AMPK-related kinases by phosphorylating the T-loop threonine residue equivalent to AMPKα1/α2 Thr^172^ located within the kinase domains of these enzymes [18]. In contrast with AMPKα1 or AMPKα2, the AMPK-related kinases do not possess adenine nucleotide, such as AMP, -binding regulatory subunits and are not stimulated by energy stress [9]. Previous studies have shown that the AMPK-related kinases play critical roles in controlling physiological processes such as polarity [19], adhesion [20], proliferation [21] and CREB (cAMP-response-element-binding protein)-mediated gene transcription [22,23].

LKB1 is phosphorylated and prenylated at a highly conserved motif within its C-terminal residues. In mice the C-terminal residues are KIRRLSACKQQ, corresponding to residues 426–436 of mouse LKB1 in which the underlined Ser^431^ residue is phosphorylated and the underlined Cys^433^ residue is farnesylated [24–26]. The farnesylated cysteine residue lies within a CAAX motif required for prenylation of all proteins [27,28]. LKB1 prenylation probably promotes association with the plasma membrane as mutation of Cys^433^ to alanine or serine, to prevent farnesylation, was shown to reduce levels of LKB1 associated with the plasma membrane in several studies [20,25,29]. Ser^431^ is phosphorylated by the PKA (cAMP-dependent protein kinase) in response to agonists that stimulate cAMP production or by the p90 RSK (ribosomal S6 kinase) in response to stimuli that trigger the activation of the ERK1 (extracellular-signal-regulated kinase 1)/ERK2 MAPKs (mitogen-activated protein kinases) [24–26,30]. The CAAX motif and the basic residues at the −2, −3 and −5 positions from Ser^431^, required for phosphorylation by PKA and p90 RSK, are evolutionarily conserved in mammals, frogs, fish and insects suggesting that these play a fundamental role. In *Caenorhabditis elegans* the orthologue of LKB1 [Par-4 (abnormal embryonic partitioning of cytoplasm 4)] possesses a residue at its C-terminus that is equivalent Ser^431^, but lacks the CAAX prenylation motif [29].

A splice variant of LKB1 termed LKB1_short_ has been identified in which the C-terminal 63 residues encompassing Ser^431^ and Cys^433^ are replaced by a unique 39-residue sequence lacking known phosphorylation and farnesylation sites [31–33]. Although LKB1_short_ is expressed in several tissues its levels are particular high in haploid spermatids in the testis. Male mice possessing reduced levels of LKB1 in all tissues, but that also lack expression of LKB1_short_, are sterile [31,33,34]. More detailed analysis revealed that these animals displayed a dramatically reduced number of mature spermatozoa in the epididymis due to a defect in spermatozoa release during spermiation [32,33].

How Ser^431^ phosphorylation or Cys^433^ prenylation affects the function of LKB1 is not understood. Mutation of Ser^431^ has no effect on the ability of LKB1 to associate with STRAD and MO25 or to phosphorylate and activate AMPK *in vitro* [6,31,35]. There is also no evidence that prenylation regulates PKA-/RSK-mediated phosphorylation or vice versa as mutation of Ser^431^ to alanine did not affect the prenylation of Cys^433^ and nor did the mutation of Cys^433^ inhibit the phosphorylation of Ser^431^ [26]. Overexpression of LKB1[S431A] together with STRAD and MO25 in HeLa cells that lack LKB1 inhibited the cell cycle to the same extent as wild-type LKB1 [36]. In contrast, roles for Ser^431^ phosphorylation have been proposed that include promoting axon specification in the developing nervous system in response to BDNF (brain-derived neurotrophic factor) by promoting the activation of BRSK1 (BR serine/threonine kinase 1)/BRSK2 (also known as SAD-B/SAD-A) [37,38]. *Drosophila* loss-of-function mutations in the *Lkb1* gene caused defects in the polarity of the oocyte, and this was rescued by low-level expression in the germ line of wild-type LKB1, but not by mutation of the residue homologous with Ser^431^ (Ser^535^) [29].

In an attempt to better define the physiological roles that C-terminal post-translational modification play we generated knockin mice in which Ser^431^ was mutated to alanine to prevent phosphorylation or Cys^433^ was mutated to serine to prevent farnesylation. Although the homozygous LKB1^S431A/S431A^ and LKB1^C433S/C433S^ mice exhibited no overt phenotypes, we found that the LKB1^C433S/C433S^ animals displayed lower levels of LKB1 at their membrane and, surprisingly, had significantly reduced basal, as well as stimulated, AMPK activity in all of the tissues and cells analysed. This provides the first evidence that the membrane association of LKB1 is required for efficient activation of AMPK *in vivo*. We discuss the possibility that myristoylation of the AMPKβ subunit and farnesylation of LKB1 may promote the interaction of these enzymes on a two-dimensional surface of the plasma membrane, which could operate to facilitate the activation of AMPK by LKB1.

## MATERIALS AND METHODS

### Materials

Complete protease inhibitor cocktail tablets were obtained from Roche, AICAR (5-amino-4-imidazolecarboxamide riboside) was from Apollo Scientific, 2-deoxy-D-[1-^3^H]glucose and D-[^14^C]mannitol were from PerkinElmer, and [γ-^32^P]ATP and Protein G–Sepharose were purchased from GE Healthcare. Phosphocellulose P81 paper was from Whatman. [1-^14^C]Acetic acid was obtained from American Radiolabeled Chemicals. Phenformin, forskolin and PMA were purchased from Sigma. All plasmids, antibodies and recombinant proteins that we have generated for the present study are available on request from our reagents website (http:s://mrcppureagents.dundee.ac.uk/).

### Antibodies

The following antibodies were raised by the DSTT (Division of Signal Transduction Therapy) at the University of Dundee in sheep and affinity-purified against the indicated antigens: anti-AMPKα1 (S524D 2nd bleed, CTSPPDSFLDDHHLTR, residues 355–369 of human AMPKα1) and anti-AMPKα2 (S525D 2nd bleed, CMDDSAMHIPPGLKPH, residues 353–366 of human AMPKα2) [10], and anti-LKB1 (S170D 2nd bleed, raised against full-length mouse LKB1), anti-SIK3 (salt-inducible kinase 3) (S226B, 3rd bleed, TDILLSYKHPEVSFSMEQAGV, residues 1349–1369 of human SIK3), anti-NUAK1 [NUAK family, SNF1-like kinase 1; also known as ARK5 (AMPK-related protein kinase 5)] (S628B, 1st bleed, raised against full-length human NUAK), anti-MARK4 (MAP/microtubule affinity-regulating kinase 4) (S272B, 3rd bleed, MSSRTVLAPGNDRNSDTHGT, residues 1–20 of human MARK4), anti-phospho-TBC1D1 [TBC (Tre-2/Bub2/Cdc16) domain family, member 1] Ser^237^, anti-TBC1D1 (S279C 1st bleed, raised against full-length human TBC1D1), anti-BRSK1 (S222B 3rd bleed, MVAGLTLGKGPESPDGDVSV, residues 1–20 of human BRSK1) and anti-BRSK2 (S223B 2nd bleed, LSWGAGLKGQKVATSYESSL, residues 655–674 of human BRSK2). The anti-HA (haemagglutinin) antibody was from Roche. The anti-LKB1 (catalogue number 3047), anti-phospho-LKB1 Ser^431^ (catalogue number 3482), anti-GAPDH (glyceraldehyde-3-phosphate dehydrogenase; catalogue number 2118), anti-HSP90 (heat-shock protein 90; catalogue number 4874), anti-Na^+^/K^+^-ATPase (catalogue number 3010), anti-phospho-AMPK Thr^172^ (catalogue number 2535), anti-AMPKα1/α2 (catalogue number 2532) anti-phospho-raptor [regulatory associated protein of mTOR (mammalian target of rapamycin)] Ser^792^ (catalogue number 2083), anti-raptor (catalogue number 2280), anti-phospho-ACC (acetyl-CoA carboxylase) Ser^79^ (catalogue number 3661), anti-ACC (catalogue number 3662), anti-NUAK1/ARK5 (catalogue number 4458), anti-lamin A/C (catalogue number 2032), anti-EEA1 (early endosome antigen 1; catalogue number 2411), anti-BiP [immunoglobulin heavy-chain-binding protein; also known as GRP78 (78 kDa glucose-related protein) catalogue number 3177], anti-LAMP1 (lysosomal-associated membrane protein 1; catalogue number 3243), anti-phospho-ERK1/2 Thr^202/^Tyr^204^ (catalogue number 4377) and anti-ERK1/2 (catalogue number 4695) were purchased from Cell Signaling Technology. Secondary antibodies coupled to HRP (horseradish peroxidase) were from Thermo Scientific. We also attempted to analyse the expression of endogenous mouse LKB1 by immunofluorescence. However, we were unable to identify an antibody that was capable of specifically localizing LKB1 expression in wild-type MEFs (mouse embryonic fibroblasts) in which no signal was observed in parallel experiments with LKB1-knockout MEFs. The antibodies tested were anti-LKB1 (catalogue number 3047, rabbit monoclonal) from Cell Signaling Technology and anti-LKB1 [Ley 37D/G6] (catalogue number 15095, mouse monoclonal) from Abcam. The protocol used was as follows. Cells for immunofluorescence studies were grown on glass coverslips. They were fixed in 4%paraformaldehyde for 10 min followed by permeabilization with 1% Nonidet P40 for 15 min. Blocking was performed with donkey serum for 1 h and the cells were then incubated with primary antibodies diluted in PBS containing 0.2% BSA and 0.02% sodium azide for 1 h at room temperature (20°C). Cells were washed with PBS containing 0.2% BSA and 0.02% sodium azide and incubated with Alexa Fluor®-conjugated secondary antibodies (raised in donkeys) for 1 h at room temperature. Cells were then washed with PBS containing 0.2% BSA and 0.02% sodium azide and the slides mounted with a mounting medium followed by visualization with a confocal microscope.

### Expression and purification of farnesyl transferase

The expression plasmid encoding the His-tagged recombinant farnesyl transferase α and β subunits was from Dr Aymelt Itzen (Max-Planck-Institute of Molecular Physiology, Dortmund, Germany). It was transformed into *Escherichia coli* BL21 cells and cultured at 37°C with shaking until a *D* value of 0.8 was achieved. Protein expression was induced with 100 μM IPTG for 4 h at 37°C with shaking. Bacteria were pelleted by centrifugation at 6500 ***g*** for 30 min at 4°C. Cells were then resuspended in 17 ml of lysis buffer (50 mM sodium phosphate monobasic, pH 8, 300 mM NaCl, 1 mM PMSF and 1 mM benzamidine) per litre of culture. Cells were lysed by sonication at 70% amplitude for 1 min on ice and the resulting supernatant was clarified by centrifugation at 31500 ***g***. The supernatant was passed over a 10 ml chelating Sepharose IDA column (GE Life Sciences) loaded with NiSO_4_. Unbound protein was washed from the column using 10 bed volumes of lysis buffer or until the UV 280 value returned to base levels. His-tagged proteins were eluted over a 0–500 mM imidazole gradient and the fractions containing the protein pooled. Following buffer exchange on a Hiprep 26/10 desalting column (GE Life Sciences) into 25 mM Hepes (pH 7.2), the pooled fraction was loaded on to a Hitrap Q column (GE Life Sciences). After washing with 5 bed volumes of the loading buffer, the protein was eluted over a 0–500 mM NaCl gradient. A final purification across a G200 Sephadex column into 25 mM Hepes (pH 7.2), 40 mM NaCl and 1 mM DTT resulted in a highly pure and active enzyme.

### Synthesis of farnesyl pyrophosphate

Farnesyl pyrophosphate was synthesized as described previously [39].

### Farnesylation and purification of the LKB1 peptide

The LKB1 peptide (CKIRRLSACKQQ) (synthesized by GL Biochem) was farnesylated at 37**°**C for 3 h in a prenylation buffer containing 25 mM Hepes (pH 8.5), 40 mM NaCl, 2 mM MgCl_2_, 20 μM ZnCl_2_, 1 mM TCEP [tris(2-carboxyethyl)phosphine], 160 μM farnesyl pyrophosphate and 5 μM farnesyl transferase.

Isolation of the non-farnesylated from the farnesylated peptide was done by SPE (solid-phase extraction) using STRATA™-C18-E polymeric reversed phase end-capped C_18_ sorbent (for strong hydrophobic retention by the active silanol groups) cartridges (Phenomenex) at 25% (non-farnesylated), 60% (farnesylated) and 100% acetonitrile/0.1% trifluoroacetic acid. The presence and purity of the peptide in the different fractions were tested by MALDI–TOF-MS (UltrafleXtreme, Bruker Daltonics) using a mixture of α-cyano-4-hydroxycinnamic acid and 2,5-dyhydroxybenzoic acid (1:1) as a matrix, in reflectron positive mode. For external calibration, a mix of nine mono-isotopic masses were used, i.e. bradykinin, [*M*+H]^+^=757.3992 Da; angiotensin II, [*M*+H]^+^=1046.5418 Da; angiotensin I, [*M*+H]^+^=1296.6848 Da; substance P, [*M*+H]^+^=1347.7354 Da; bombesin, [*M*+*H*]^+^=1619.8223 Da; renin substrate, [*M*+H]^+^=1758.9326 Da; ACTH (adrenocorticotropic hormone) clip 1–17, [*M*+H]^+^=2093.0862 Da; ACTH clip 18–39, [*M*+H]^+^=2465.1983 Da; and somatostatin 28, [*M*+H]^+^=3147.4710 Da (Bruker Daltonics).

To assess the efficiency of the reaction and thus the purity of the peptide after the farnesylation reaction, the product was run on an off-line HPLC Ultimate 3000 system (Thermo Scientific) and the chromatograms were acquired using a VWD-3400RS UV–Vis photometer at a wavelength of 214 nM. Samples were run on a highly resolving C_18_ column (Gemini-C18, 3 μm, 110Å, 3 mm×250 mm, Phenomenex) and peptide separation was achieved with a flow rate of 0.20 ml/min (solvent A, 0.1% trifluoroacetic acid and solvent B, acetonitrile/0.08% trifluoroacetic acid). A gradient (slope 5) spanning 0–100% mobile phase B over 130 min was used.

### Production and purification of an LKB1 farnesylation-specific antibody

The purified LKB1 farnesylated peptide (10 mg) was used by Dundee Cell Products to produce a mouse monoclonal antibody that strongly recognized this antigen. The hybridoma expressing the most sensitive immunoblotting antibody that recognized farnesylated LKB1 was selected and antibody purified accordingly to the company's protocol.

### Generation and genotyping of LKB1^C433S/C433S^ and LKB1^S431A/S431A^ mice

TaconicArtemis generated the LKB1^C433S/C433S^ and LKB1^S431A/S431A^ mice. The mice were generated and maintained on a C57BL/6J background. Genotyping was performed by PCR using genomic DNA isolated from ear biopsies. Primer 1 (5′-CTAGTGTGGC-CAAGTCAGAGG-3′) and primer 2 (5′-AGACCAGCTTGCTC-TGTTGG-3′) were used to detect the wild-type and knockin alleles. The PCR program consisted of 5 min at 95**°**C, then 35 cycles of 30 s at 95**°**C, 30 s at 60**°**C, 1 min at 72**°**C and 10 min at 72**°**C.

All animal studies and breeding was approved by the University of Dundee ethical committee and performed under a U.K. Home Office project licence.

### Intraperitoneal glucose- and AICAR-tolerance test

Mice were fasted for 6 h and injected intraperitoneally with glucose (2 g/kg of body mass) or AICAR (250 mg/kg of body mass) diluted in PBS. Blood was collected from the tail tip before and at various times after injection. Blood glucose levels were measured by an AlphaTrak glucometer (Abbott Laboratories).

### Generation of MEFs and stimulations

LKB1^C433S/C433S^ and LKB1^S431A/S431A^ MEFs isolated from mouse embryos at E13.5 (embryonic day 13.5) were generated as described previously [40] and immortalized by continuous passaging. Cells were cultured in DMEM (Dulbecco's modified Eagle's medium) containing 10% FBS (Sigma), 2 mM L-glutamine, 50 units/ml penicillin G and 50 μg/ml streptomycin (Life Technologies). Cells were treated with AICAR (2 mM) and phenformin (2 mM) for 1 h. Cells were washed with PBS and lysed in ice-cold lysis buffer. Lysates were clarified by centrifugation at 13000 ***g*** for 15 min at 4**°**C, and the supernatant was snap-frozen and stored at −80**°**C. The lysates (20 μg) in SDS sample buffer were then subjected to immunoblotting.

LKB1^+/+^ and LKB1^−/−^ MEFs, provided by Professor Tomi Mäkelä (University of Helsinki, Helsinki, Finland) and described previously [10], were cultured in DMEM containing 10% FBS, 2 mM L-glutamine, 50 units/ml penicillin G and 50 μg/ml streptomycin. Cells were stimulated with forskolin (20 μM) for 10 min or PMA (400 ng/ml) for 20 min. For lysis, cells were washed with PBS and lysed in ice-cold lysis buffer. Lysates were centrifuged at 13000 ***g*** for 15 min at 4**°**C, and the supernatant was snap-frozen and stored at −80**°**C. The lysates (20 μg) in SDS sample buffer were then subjected to immunoblotting.

### Transfection of HEK (human embryonic kidney)-293 cells

HEK-293 cells were transfected with a pCMV5-encoded DNA construct expressing the plasmids indicated in the Figure legends. At 36 h post-transfection cells were washed with PBS and lysed in ice-cold lysis buffer. Lysates were centrifuged at 13000 ***g*** for 15 min at 4**°**C, and the supernatant was snap-frozen and stored at −80**°**C. The lysates (20 μg) in SDS sample buffer were then subjected to immunoblotting.

### Subcellular fractionation of mouse livers and MEFs

Mouse liver (50 mg) or 20 μl of packed MEFs were subjected to subcellular fractionation using commercial kits (catalogue numbers 87790 and 78840, Thermo Scientific). Cytoplasmic, membrane and soluble nuclear fractions were prepared accordingly to the manufacturer's protocol using the buffers provided. The amount of protein in each fraction was quantified using the Bradford assay and then subjected to immunoblot analysis.

### Primary hepatocyte isolation and treatment

Mouse primary hepatocytes were isolated as described previously [41]. Isolated primary hepatocytes were plated in M199 medium (Invitrogen) containing 10% FBS (Sigma), 0.1% BSA (Invitrogen), 10 nM insulin (Novo-Nordisk), 200 nM tri-iodothyronine (Sigma), 500 nM dexamethasone (Sigma), 50 units/ml penicillin G and 50 μg/ml streptomycin (Life Technologies) and then left to attach for 4 h at 37**°**C in a 5% CO_2_ atmosphere. The medium was then replaced for serum-free M199 medium containing 100 nM dexamethasone. The next day, cells were treated with an increased concentration of AICAR for 1 h, lysed and submitted to immunoblotting.

### Preparation of tissue lysates

Mouse tissues were rapidly excised, frozen in liquid nitrogen and stored at −80**°**C. Tissues were subsequently homogenized on ice using a Kinematica Polytron in a 10-fold mass excess of ice-cold lysis buffer containing 50 mM Tris/HCl (pH 7.5), 1 mM EDTA, 1 mM EGTA, 1% Triton X-100, 1 mM sodium orthovanadate, 50 mM NaF, 5 mM sodium pyrophosphate, 0.27 M sucrose, 0.1% 2-mercaptoethanol and Complete protease inhibitor cocktail. Tissues lysates were clarified by centrifugation at 13000 ***g*** for 15 min at 4**°**C. Supernatants were removed, snap-frozen and stored at −80**°**C.

### Incubation of isolated muscle with AICAR

Mice were killed by cervical dislocation and their EDL (extensor digitorum longus) muscles were rapidly removed. Tendons from both ends of each muscle were tied with suture and mounted on an incubation apparatus. The muscles were incubated with 2 mM AICAR as described previously [42].

### *In situ* muscle contraction

Mice were anaesthetized with sodium pentobarbital (90 mg/kg of body mass, administrated intraperitoneally), the sciatic nerves of both legs were surgically exposed and electrodes were attached. Muscle contraction was performed as described previously [34].

### Glucose transport in isolated skeletal muscle

Mice were killed by cervical dislocation and their EDL muscles were rapidly removed. Tendons from both ends of each muscle were tied with suture, mounted on an incubation apparatus and incubated as previously described to study the effect of AICAR stimulation on glucose uptake [34]. To study the effect of *in situ* muscle contraction on glucose uptake, sciatic nerve stimulation was performed as in [34], the EDL muscle was isolated and then 2-deoxy-glucose uptake was measured.

### Immunoblotting

Tissues or cell lysates (20 μg) were heated in SDS sample buffer and subjected to SDS/PAGE (8 or 10% gel) followed by electrotransfer on to nitrocellulose membranes. Membranes were blocked in TBST [50 mM Tris/HCl (pH 7.5), 0.15 mM NaCl and 0.1% Tween] containing 5% non-fat dried skimmed milk powder for 1 h. The membranes were then probed with the primary antibody (1 μg/ml for the sheep antibodies or 1000-fold dilution for the commercial antibodies) for 16 h at 4**°**C in TBST containing 5% non-fat dried skimmed milk powder (sheep antibodies) or 5% BSA (commercial antibodies). Detection of protein was performed using HRP-conjugated secondary antibodies and an ECL reagent. Increasing amounts of non-farnesylated and farnesylated LKB1 peptides were spotted on to nitrocellulose membrane and then probed for 16 h with the anti-LKB1 farnesylation-specific antibody or anti-LKB1 antibodies at 4**°**C in TBST containing 5% non-fat dried skimmed milk powder (sheep antibodies) or 5% BSA (commercial antibodies). Detection of protein was performed using HRP-conjugated secondary antibodies and an ECL reagent. Quantitative immunoblot analysis was performed by Li-Cor analysis. Briefly, blots were processed as above, but incubated with a fluorescent secondary antibody allowing for detection with the Li-Cor Odyssey infrared system. Band intensity was quantified using Li-Cor software.

### Immunoprecipitation and kinase activity

Tissues or cell lysates (0.05–2 mg) were incubated at 4**°**C for 1 h on a shaking platform with 5 μl of protein G–Sepharose coupled to anti-LKB1, anti-AMPKα1, anti-AMPKα2, anti-SIK3, anti-NUAK1, anti-MARK4, anti-BRSK1 and anti-BRSK2 (sheep) antibodies. The immunoprecipitates were washed twice with 1 ml of lysis buffer containing 0.5 M NaCl and twice with buffer A [50 mM Tris/HCl (pH 7.5), 0.1 mM EGTA and 0.1% 2-mercaptoethanol]. Phosphotransferase activity towards the LKBtide peptide (SNLYHQGKFLQTFCGSPLYRRR) for LKB1, AMARA peptide (AMARAASAAALARRR) for AMPKα1 and AMPKα2, or Sakamototide substrate (ALNRTSSDSALHRRR) [43] for SIK3, NUAK1, MARK4 and BRSK1/BRSK2, were measured in a total assay volume of 50 μl consisting of 50 mM Tris/HCl (pH 7.5), 0.1 mM EGTA, 0.1% 2-mercaptoethanol, 10 mM magnesium acetate, 0.1 mM [γ^32^P]ATP and 200 μM LKBtide peptide, AMARA peptide or Sakamototide substrate. The assays were carried out at 30**°**C with continuous shaking to keep the immunoprecipitates in suspension and were terminated after 20 min by applying 40 μl of the reaction mixture on to p81 paper. These were washed in phosphoric acid and the incorporated radioactivity was measured by scintillation counting. One milliunit of activity was defined as that which catalysed the incorporation of 1 pmol of ^32^P into the substrate per min. To assess LKB1 activity by measuring the activation of an AMPK complex, endogenous LKB1 was immunoprecipitated from mouse tissue lysates (0.5 mg) as described above and incubated with 0.3 μg of dephosphorylated inactive *E. coli*-expressed recombinant AMPK complex (α1β2γ1 subunits) as described previously [8].

### Measurement of lipid synthesis from [^14^C]acetate

Primary hepatocytes were seeded in six-well plates. After an overnight incubation in serum-free M199 medium containing 100 nM dexamethasone, hepatocytes were labelled for 1 h with 1 mM acetate and 0.5 μCi/ml [1-^14^C]acetic acid in the presence or absence of increasing concentrations of AICAR. Cells were washed three times with ice-cold PBS and then scraped into chilled PBS. Primary hepatocytes were pelleted by centrifugation at 2000 ***g*** for 2 min. A methanol/chloroform lipid extraction was performed on the cell pellets. After the extraction, the insoluble material (pellet) was dissolved in 0.5 M NaOH/0.5% SDS in order to quantify the amount of protein per well. The solvent found in the supernatant was evaporated and the incorporation of [^14^C] into the lipid was measured by scintillation counting. The incorporation rate was defined in units of μmol of acetate/g of protein per h.

### Identification of LKB1-interacting protein by MS

Tissue lysates (50 mg) were pre-cleared by incubation with 100 μl of pre-immune IgG covalently coupled to Protein G–Sepharose for 1 h at 4**°**C on a rolling shaker. The supernatants were then incubated with 50 μg of anti-LKB1 farnesylation-specific antibody covalently coupled to Protein G–Sepharose for 1 h at 4**°**C on a rolling shaker. The immunoprecipitates were washed three times with 10 ml of lysis buffer containing 0.5 M NaCl and twice with 10 ml of buffer A. The beads were resuspended in a total volume of 30 μl of LDS sample buffer (Invitrogen). The samples were then filtered with a 0.44 μm Spin-X filter (Corning), reduced with 10 mM DTT, boiled and subjected to electrophoresis on a NuPAGE Bis-Tris 4–12% polyacrylamide gel. Colloidal Coomassie-stained gels were divided and each piece was washed with 0.1 M NH_4_HCO_3_ and 50% acetonitrile/50 mM NH_4_HCO_3_, alkylated with 50 mM iodoacetamide in 0.1 M NH_4_HCO_3_ (30 min at room temperature), washed as above, dried, and incubated with 25 mM triethylammonium bicarbonate with 5 μg/ml trypsin overnight at 30**°**C on a shaker. The resulting peptides were submitted to LC–MS on a Proxeon EASY-nLC nano-LC system coupled to a Thermo-LTQ-Orbitrap mass spectrometer. Data files were searched against the SwissProt mouse database using Mascot (http://www.matrixsciences.com) run on an in-house system, with a 10 p.p.m. mass accuracy for precursor ions, a 0.6 Da tolerance for fragment ions, and allowing for carbamidomethyl (C) as a fixed modification and for oxidation and dioxidation (M) as variable modifications.

## RESULTS

### Generation of a monoclonal antibody recognizing farnesylated LKB1

**Figure 1:**
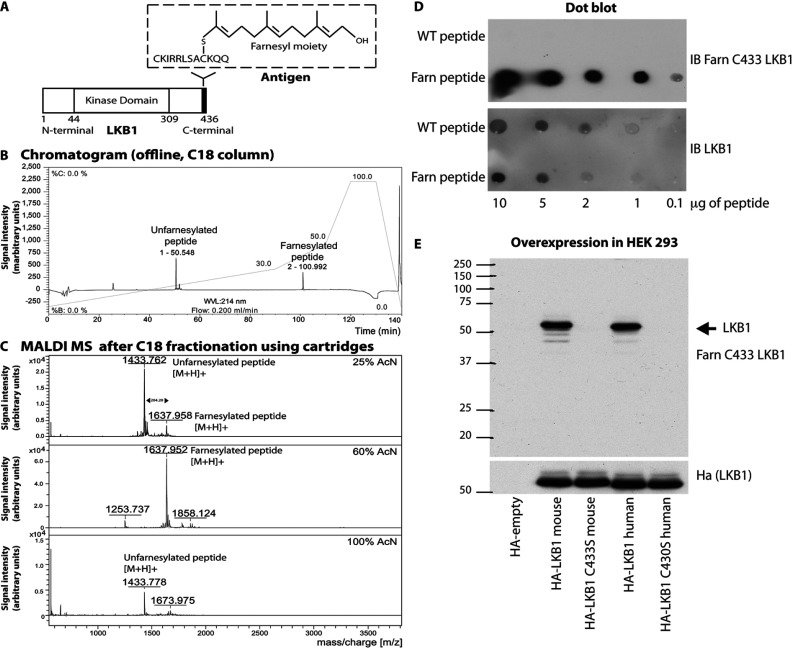
Generation of a monoclonal antibody recognizing farnesylated LKB1 (**A**) Diagram illustrating the structure of the mouse LKB1 protein as well as the peptide used for the antibody production (antigen) with the farnesyl moiety attached to the C-terminal cysteine (Cys^433^). (**B**) HPLC chromatogram and (**C**) MALDI–TOF-MS of the peptide containing Cys^433^ before and after the farnesylation reaction. (**D**) Dot-blot analysis demonstrating that the anti-LKB1 farnesylated at Cys^433^ antibody does not recognize the C-terminal cysteine on the wild-type (WT) peptide (non-farnesylated peptide). Farn peptide, farnesylated peptide; IB, immunoblot. (**E**) HEK-293 cells were transfected with the indicated mouse and human HA–LKB1 mutants. The samples were immunoblotted with the anti-LKB1 farnesylated at Cys^433^ antibody to ensure that the antibody does not recognize the cysteine mutant proteins. Molecular mass is given on the left-hand side in kDa.

To generate an antibody that recognized farnesylated LKB1, we prenylated a peptide encompassing the C-terminal motif of LKB1 using recombinant farnesyl transferase and farnesyl pyrophosphate (Figure 1A). The prenylated peptide was purified by chromatography, characterized by HPLC and MALDI–TOF-MS (Figures 1B and 1C) and used to raise a mouse monoclonal antibody. A hybridoma was identified that strongly recognized the prenylated peptide, but not the non-prenylated peptide (Figure 1D). This antibody recognized overexpressed wild-type human and mouse LKB1, but not mutants in which the farnesylation was prevented by mutating Cys^433^ to serine (Figure 1E).

### Evidence that the bulk of endogenous LKB1 is prenylated

To investigate whether the majority of endogenous LKB1 was prenylated, we undertook immunoprecipitation studies using the anti-LKB1 farnesylation-specific antibody on samples from mice muscle, liver, brain and testis as well as MEFs. These experiments were analysed by immunoblot analysis (Supplementary Figure S1 at http://www.biochemj.org/bj/458/bj4580041add.htm) or MS (Supplementary Table S1 at http://www.biochemj.org/bj/458/bj4580041add.htm). This revealed that the anti-LKB1 farnesylation-specific antibody immunoprecipitated the vast majority of endogenous LKB1 from all tissues/MEF extracts examined. Immunoblot analysis of the supernatant of anti-LKB1 farnesylation-specific antibody immunoprecipitates revealed that a relatively small portion of LKB1 that was not immunoprecipi-tated was still prenylated. Analysing the anti-LKB1 farnesylation-specific antibody immunoprecipitates by MS confirmed that LKB1 and its regulatory STRAD and MO25 subunits were co-immunoprecipitated. Moreover, in addition to LKB1, 13 other proteins with C-terminal CAAX motifs, that are therefore likely to be farnesylated, were immunoprecipitated including MAPKAPK3 [MAPKAP (MAPK-activated protein) kinase-3] that, to our knowledge, has not been previously reported to be farnesylated (Supplementary Tables S1 and S2 at http://www.biochemj.org/bj/458/bj4580041add.htm). The anti-LKB1 farnesylation-specific antibody may also recognize geranylgeranylated prenylated proteins, as we found at least eight Rab GTPase isoforms co-immunoprecipitating with the anti-LKB1 farnesylation-specific antibody that are modified by introduction of two geranylgeranyl groups on to two cysteine residues at the C-terminal consensus sequence, XCXC or XXCC, which is introduced by the Rab geranylgeranyl transferase [44] (Supplementary Tables S1 and S2). A sequence alignment of the six C-terminal residues of each prenylated protein co-immunoprecipi-tated with the anti-LKB1 farnesylation-specific antibody is also shown in Supplementary Table S2.

### Characterization of the farnesylation-deficient LKB1^C433S/C433S^ mouse

**Figure 2:**
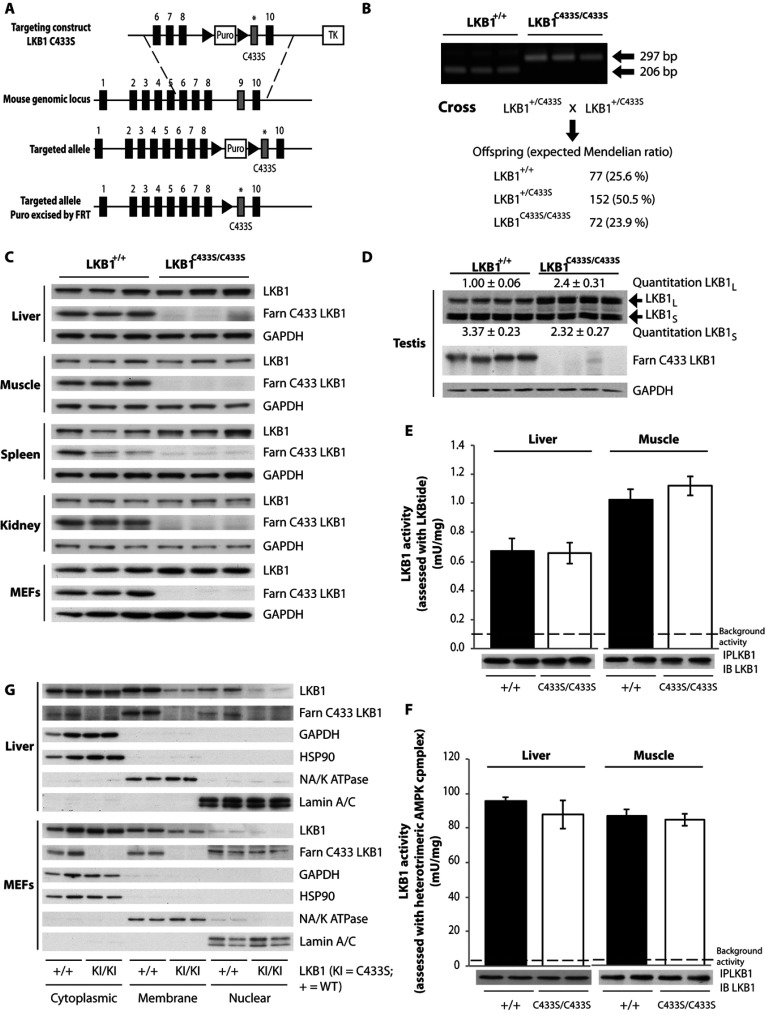
Characterization of the farnesylation-deficient LKB1^C433S/C433S^ mouse (**A**) The knockin construct, the endogenous LKB1 allele containing exons 1–10 and the target allele with the puromycin cassette (Puro) removed by Flp (flippase) recombinase are depicted. The black/grey rectangles represent exons and the black triangles represent FRT (flippase-recognition target) sites. TK, thymidine kinase. (**B**) To genotype mice, genomic DNA was PCR-amplified. Sizes are indicated in bp. The breeding strategy employed to generate LKB1^C433S/C433S^ mice is shown with the number and percentage of each genotype obtained indicated. (**C**) Tissues lysates from wild-type LKB1^+/+^ and LKB1^C433S/C433S^ mice and MEFs were subjected to immunoblotting using anti-LKB1 and anti-LKB1 farnesylation-specific (LKB1 Farn C433) antibodies. (**D**) Testis lysates from LKB1^+/+^ and LKB1^C433S/C433S^ mice were immunoblotted with an anti-LKB1 antibody to detect the LKB1_long_ (LKB1_L_) and LKB1_short_ (LKB1_s_) isoforms. Band intensities were quantified using Li-Cor. Results are means±S.E.M. for four mice per genotype. (**E**) LKB1 was immunoprecipitated (IP) from the liver and muscle (EDL) from wild-type LKB1^+/+^ (+/+) and LKB1^C433S/C433S^ (C433S/C433S) mice and the *in vitro* kinase activity towards the LKBtide peptide was measured. Immunoprecipitates were also immunoblotted (IB). Assays were performed in duplicate from tissues taken from six mice per genotype and the results are means±S.E.M. The broken line represents the background activity as measured with pre-immune IgG. (**F**) As (**E**), except that the immunoprecipitated LKB1 protein was used to activate a recombinant heterotrimeric AMPK complex (α1β2γ1) and then AMPK kinase activity towards the AMARA peptide was measured. Assays were performed in duplicate from tissues taken from six mice per genotype and the results are means±S.E.M. (**G**) Liver and MEFs from wild-type (WT) LKB1^+/+^ (+/+) and LKB1^C433S/C433S^ (KI/KI) mice were submitted to subcellular fractionation. Cytoplasmic, membrane and nuclear fractions were immunoblotted with the indicated antibodies. Blots for two animals out of four per genotype are shown.

Knockin mice in which the LKB1 farnesylated Cys^433^ is changed to serine to abolish prenylation were generated and maintained on an inbred C57BL/6J background as shown in Figure 2(A). Homozygous LKB1^C433S/C433S^ mice were born at the expected Mendelian frequency (Figure 2B), were of normal size and appearance, and did not display any overt phenotype at least up to 1 year of age (the oldest animals we have analysed). Immunoblot analysis using the anti-LKB1 farnesylation-specific antibody confirmed that LKB1 was no longer prenylated in tissues or MEFs derived from the LKB1^C433S/C433S^ mice (Figures 2C and 2D). Immunoprecipitation studies with the anti-LKB1 farnesylation-specific antibody also confirmed that LKB1, STRAD and MO25 were immunoprecipitated from various tissues (brain, liver, muscle and testis) and MEFs from wild-type, but not LKB1^C433S/C433S^, mice in experiments conducted in parallel (Supplementary Figure S1 and Supplementary Table S1 at http://www.biochemj.org/bj/458/bj4580041add.htm). Total LKB1 immunoblot analysis also revealed that the LKB1[C433S] mutant was expressed at similar levels to the wild-type LKB1 in the liver, muscle, spleen, kidney and MEFs (Figure 2C).

### Farnesylation of LKB1 in the testis

We also immunoblotted testis, which, as mentioned in the Introduction section, expresses a high level of the LKB1_short_ isoform that lacks the C-terminal prenylation motif. Immunoblot analysis revealed that the levels of LKB1_short_ were not affected significantly in the testis of LKB1^C433S/C433S^ mice (Figure 2D). Immunoprecipitation studies with the anti-LKB1 farnesylation-specific antibody confirmed that the normal LKB1_long_ isoform was immunoprecipitated, but not the LKB1_short_ isoform, which is not farnesylated [31–33] (Supplementary Figure S1). However, in contrast with other tissues, we found that the levels of the LKB1_long_ isoform were expressed at approximately 2-fold higher levels in the testis of LKB1^C433S/C433S^ mice compared with their littermate wild-type mice (Figure 2D). The reasons for this are not clear and require further investigation. The LKB1^C433S/C433S^ mice are fertile as crosses of homozygous LKB1^C433S/C433S^ animals resulted in viable offspring.

### Farnesylation does not influence LKB1 catalytic activity

To assess how the C433S mutation affected the kinase activity of LKB1 we immunoprecipitated endogenous LKB1 from the muscle and liver and studied its kinase activity employing either the LKBtide peptide substrate, derived from the NUAK2 T-loop motif [18] (Figure 2E) or a recombinant full-length heterotrimeric AMPKα1–AMPKβ2–AMPKγ1 complex expressed in *E. coli* cells (Figure 2F). This revealed that whichever way LKB1 catalytic activity was assessed, loss of farnesylation had no effect on catalytic activity.

### Evidence that farnesylation of LKB1 promotes membrane localization

To assess whether loss of LKB1 farnesylation affected membrane localization, we fractionated liver and MEF (Figure 2G, upper and lower panel respectively) extracts, derived from littermate wild-type and LKB1^C433S/C433S^ knockin mice, into cytoplasmic, membrane and nuclear fractions and analysed the relative levels of LKB1 by immunoblot analysis. Consistent with previous findings [20,25,29] in samples derived from wild-type mice, significant levels of LKB1 were present in the cytoplasmic and membrane fractions with only low levels of LKB1 observed in the nuclear fractions. Immunoblotting with the anti-LKB1 farnesylation-specific antibody revealed that the LKB1 present in all fractions was farnesylated and, consistent with the bulk of LKB1 being prenylated, the relative signal observed paralleled the total expression of LKB1. However, consistent with the notion that the farnesylation of LKB1 promotes association with membranes, we observed that the levels of LKB1 in the membrane fractions were significantly reduced in the livers and MEFs of LKB1^C433S/C433S^ mice compared with the wild-type (Figure 2G). We also observed a reduction in non-farnesylated LKB1 in the nuclear fraction of the livers and MEFs of LKB1^C433S/C433S^ mice compared with the wild-type (Figure 2G). It should be noted that the fractions labelled cytoplasmic in Figure 2(G) also contained endomembranes as assessed by immunoblot analysis of ER (endoplasmic reticulum), endosome and lysosome markers (Supplementary Figure S2 at http://www.biochemj.org/bj/458/bj4580041add.htm). Moreover, the fraction labelled membrane was also contaminated with ER as identified using immunoblot analysis with a GRP78/BiP marker (Supplementary Figure S2). We also attempted to analyse the expression of endogenous mouse LKB1 by immunofluorescence. However, we were unable to identify an antibody that was capable of specifically localizing LKB1 expression in wild-type MEFs in which no signal was also observed in the LKB1-knockout MEFs examined in parallel experiments (a list of the antibodies tested is provided in the Materials and methods section).

### Impaired activity of AMPK and lipid synthesis in primary hepatocytes derived from LKB1^C433S/C433S^ mice

**Figure 3:**
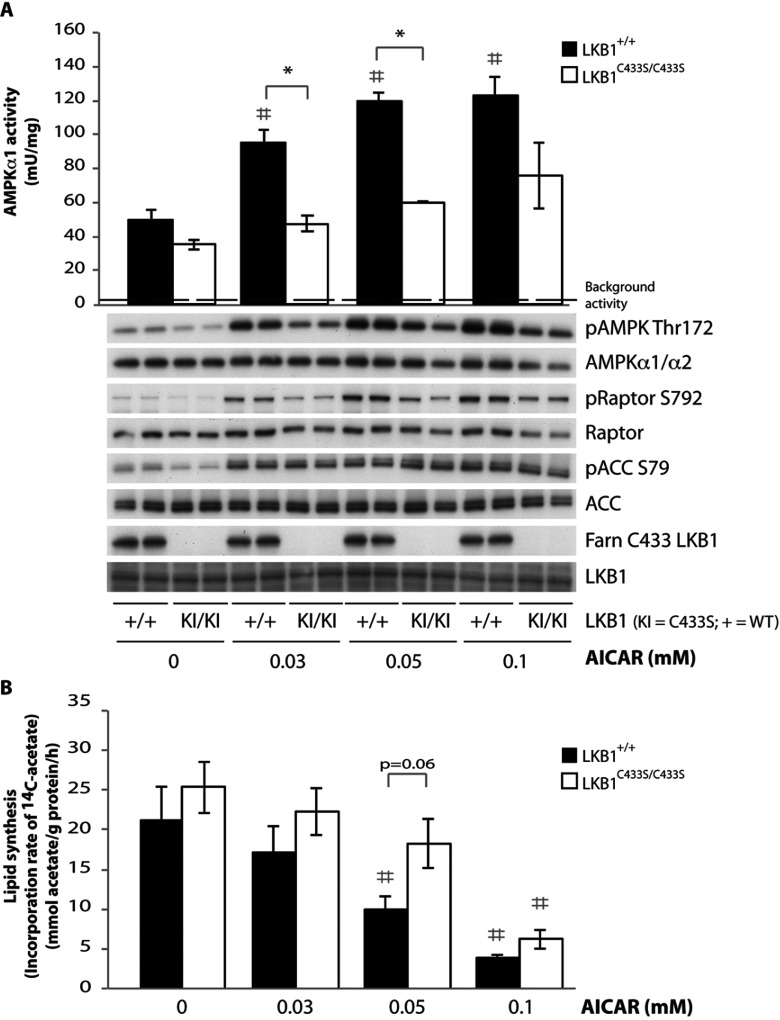
Impaired activity of AMPK and lipid synthesis in primary hepatocytes derived from LKB1^C433S/C433S^ mice Primary hepatocytes were isolated from wild-type LKB1^+/+^ (+/+) and LKB1^C433S/C433S^ (KI/KI) mice and treated with increasing concentrations of AICAR for 1 h. (**A**) Upper panel: AMPKα1 was immunoprecipitated from primary hepatocytes extracts and the *in vitro* kinase activity towards the AMARA peptide was measured. Assays were performed in duplicate for each condition and results are means±S.E.M. for three independent experiments (*n*=3). For the non-stimulated conditions the *P* value of the data for LKB1^+/+^ compared with LKB1^C433S/C433S^ mice was 0.1 and therefore judged not to be significant. The broken line represents the background activity as measured with pre-immune IgG. Lower panel: primary hepatocyte extracts were immunoblotted with the indicated antibodies. Representative immunoblots for three independent experiments (*n*=3) are shown. LKB1 Farn C433, anti-LKB1 farnesylation-specific antibody. (**B**) Lipogenesis following AICAR treatment in primary hepatocytes was assessed by using [^14^C]acetate incorporation. Assays were performed in triplicate for each condition and results are means±S.E.M. for four independent experiments (*n*=4). *P<0.05 LKB1^+/+^ compared with LKB1^C433S/C433S^ mice within each condition and #*P*<0.05 treatment compared with non-treatment condition. Statistical analysis was performed using one-way ANOVA and Tukey's post-hoc test.

To establish whether loss of LKB1 farnesylation affected the activity of AMPK, we isolated primary hepatocytes from LKB1^C433S/C433S^ and littermate wild-type mice. These were left either unstimulated or treated with increasing doses of AICAR [45], a compound that is metabolized within the cells to ZMP (5-aminoimidazole-4-carboxamide-1-β-D-furanosyl 5′-monophosphate), an analogue of AMP capable of stimulating AMPK via an LKB1-dependent mechanism [10,11]. Under basal conditions, we unexpectedly observed that AMPK activity and phosphorylation of its T-loop Thr^172^ residue (phosphorylated by LKB1) was reduced in the LKB1^C433S/C433S^ hepatocytes compared with the wild-type (Figure 3A). In hepatocytes from the wild-type animals, AICAR induced a robust activation of AMPK that was accompanied by increased phosphorylation of AMPK at Thr^172^. In hepatocytes derived from LKB1^C433S/C433S^ mice, AICAR treatment only partially stimulated AMPK activity and T-loop phosphorylation compared with the wild-type (Figure 3A). Consistent with this, phosphorylation of raptor, a known *bona fide* AMPK substrate [46], at Ser^792^ was also significantly repressed in AICAR-stimulated LKB1^C433S/C433S^ hepatocytes (Figure 3A). In contrast, monitoring the phosphorylation of ACC we observed no significant difference between the wild-type and LKB1^C433S/C433S^ knockin hepatocytes. AICAR treatment did not affect LKB1 farnesylation in wild-type hepatocytes (Figure 3A).

An established physiological consequence of AMPK activation in hepatocytes is to inhibit lipid synthesis [47]. Consistent with this notion, reduced activation of AMPK in LKB1^C433S/C433S^ hepatocytes in response to AICAR resulted in blunted AICAR-mediated suppression of lipid synthesis compared with wild-type hepatocytes (Figure 3B).

### Impaired activity of AMPK in muscle from LKB1^C433S/C433S^ mice

**Figure 4:**
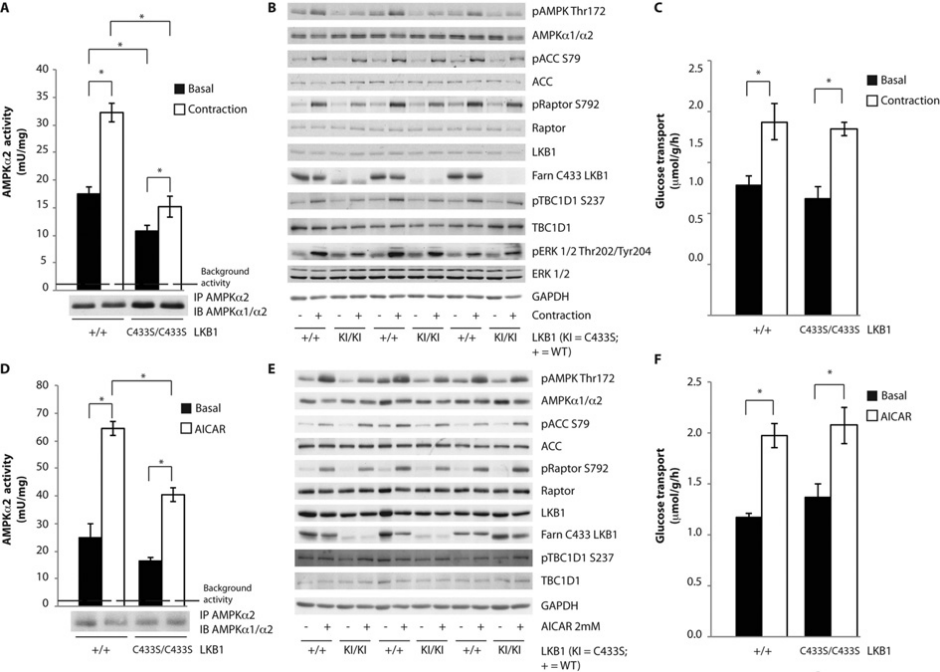
Impaired activity of AMPK in muscles taken from LKB1^C433S/C433S^ mice (**A**) One leg from anaesthetized wild-type LKB1^+/+^ (+/+) and LKB1^C433S/C433S^ (C433S/C433S) mice was subjected to *in situ* hindlimb contraction (Contraction) via sciatic nerve stimulation for 5 min and the other leg served as a non-contracted control (Basal). AMPKα2 was immunoprecipitated (IP) from tibialis anterior lysates and the *in vitro* kinase activity towards the AMARA peptide was measured. The immunoprecipitates were also immunoblotted (IB). Assays were performed in duplicate from lysates derived from five mice per genotype and results are means±S.E.M. The broken line represents the background activity as measured with pre-immune IgG. (**B**) Tibialis anterior muscle lysates were submitted to immunoblotting with the indicated antibodies. A total of three animals out of five per genotype are shown (*n*=5). (**C**) Following contraction, EDL muscles were isolated and glucose transport was measured. Results are means±S.E.M. (*n*=4–7 per group). (**D**) Isolated EDL muscle taken from wild-type LKB1^+/+^ (+/+) and LKB1^C433S/C433S^ (C433S/C433S) mice were incubated in the presence or absence of 2 mM AICAR for 50 min. AMPKα2 was immunoprecipitated from EDL lysates and the *in vitro* kinase activity towards the AMARA peptide was measured. The immunoprecipitates were also immunoblotted. Assays were performed in duplicate from lysates derived from four to five mice per genotype and results are means±S.E.M. The broken line represents the background activity as measured with pre-immune IgG. (**E**) EDL muscle lysates were submitted to immunoblotting with the indicated antibodies. A total of three animals out of five per genotype are shown (*n*=5). (**F**) Glucose transport in isolated EDL muscle was measured. Results are means±S.E.M. (*n*=4–7 per group). *P<0.05 basal compared with contraction or AICAR stimulation within each genotype. Statistical analysis was performed using one-way ANOVA and Tukey's post-hoc test.

We next assessed the level of AMPK activity and T-loop phosphorylation in skeletal muscle of littermate wild-type and LKB1^C433S/C433S^ mice. Muscle AMPK was stimulated by *in situ* contraction evoked via electrical stimulation of the sciatic nerve for one leg and the other leg served as the sham-operated non-stimulated control. As in unstimulated or AICAR-stimulated hepatocytes (Figure 3), we observed a significantly reduced basal, as well as contraction-induced, AMPK activity (Figure 4A) and T-loop phosphorylation (Figure 4B) in muscle taken from LKB1^C433S/C433S^ compared with wild-type mice. This reduced AMPK activation was also accompanied by a decreased phosphorylation of the AMPK substrates ACC [48], raptor [46] and TBC1D1 [49] at residues that are phosphorylated by AMPK. Phosphorylation of the ERK1 and ERK2 protein kinases induced by contraction [50], which is not regulated via AMPK, was not affected by the LKB1^C433S/C433S^ mutation (Figure 4B).

One of the major consequences of AMPK activation in skeletal muscle is to stimulate glucose uptake via translocation of GLUT4 (glucose transporter type 4) to the plasma membrane [51]. Previous work has shown that this translocation is very sensitive to AMPK, and even very low levels of AMPK activation are sufficient to induce maximal glucose uptake [34,52]. This probably explains why, despite reduced activation of AMPK in muscle from LKB1^C433S/C433S^ mice, contraction-induced glucose uptake was unaffected (Figure 4C). Similarly, we observed that AICAR treatment of isolated EDL muscle from LKB1^C433S/C433S^ mice resulted in a significant reduction in AMPK activation (Figure 4D) and T-loop and substrate phosphorylation compared with muscle from the wild-type mice (Figure 4E) without affecting glucose uptake (Figure 4F).

We also subjected 2-month-old LKB1^C433S/C433S^ mice to glucose- and AICAR-tolerance tests, which revealed that these animals did not display significant intolerance compared with the wild-type mice (Supplementary Figure S3 at http://www.biochemj.org/bj/458/bj4580041add.htm). Muscle contraction did not affect the farnesylation of LKB1 in muscle from wild-type mice (Figures 4B and 4E). We also generated MEFs derived from LKB1^C433S/C433S^ mice and observed a reduced activation of AMPK in basal, as well in AICAR- and phenformin-treated, cells (Supplementary Figure S4 at http://www.biochemj.org/bj/458/bj4580041add.htm).

### Characterization of the LKB1^S431A/S431A^ mouse

**Figure 5:**
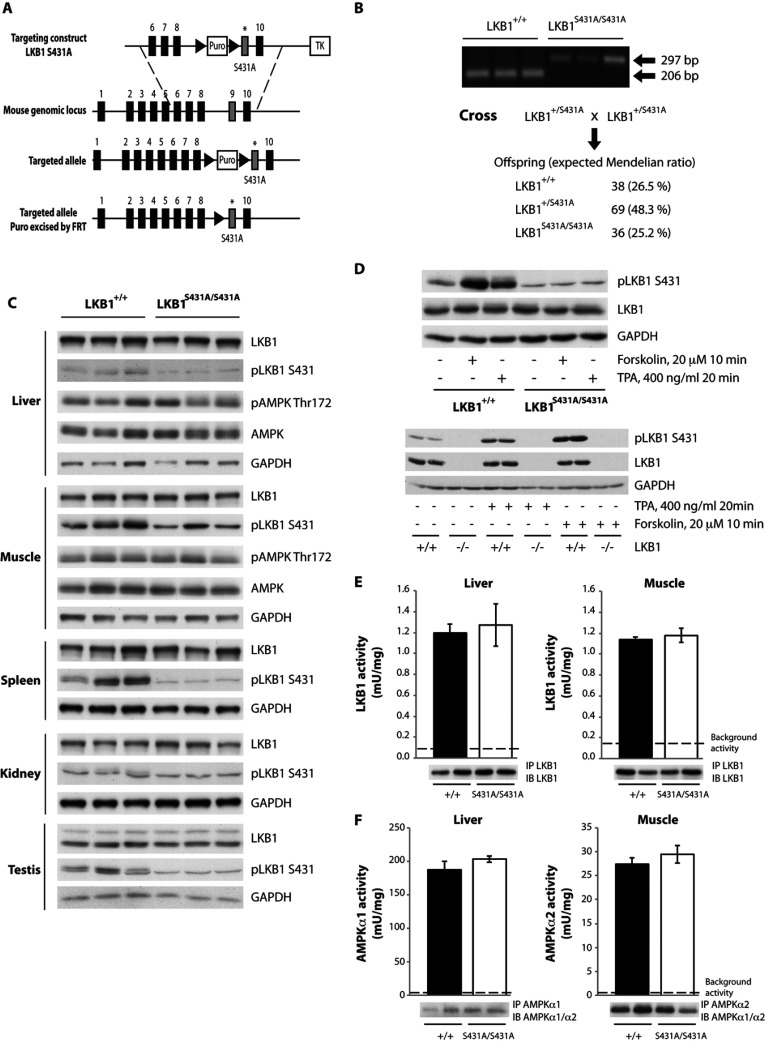
Characterization of the LKB1^S431A/S431A^ mouse (**A**) The knockin construct, the endogenous *LKB1* allele containing exons 1–10 and the target allele with the puromycin cassette (Puro) removed by Flp recombinase are shown. The black/grey rectangles represent exons and the black triangles represent FRT (flippase-recognition target) sites. TK, thymidine kinase. (**B**) To genotype mice, genomic DNA was PCR-amplified. Sizes are indicated in bp. The breeding strategy employed to generate LKB1^S431A/S431A^ mice is shown with the number and percentage of each genotype obtained indicated. (**C**) Tissues lysates from wild type LKB1^+/+^ and LKB1^S431A/S431A^ mice were subjected to immunoblotting using anti-LKB1 and anti-phospho-LKB1 Ser^431^ antibodies. (**D**) MEFs derived from wild-type LKB1^+/+^ and LKB1^S431A/S431A^ mice (upper panel) or wild type LKB1^+/+^ (+/+) and knockout LKB1^−/−^ (−/−) mice (lower panel) were stimulated with forskolin (20 μM for 10 min) or TPA (400 ng/ml for 20 min) and immunoblotted with the indicated antibodies. (**E**) LKB1 was immunoprecipitated (IP) from the liver and muscle (EDL) from wild-type LKB1^+/+^ (+/+) and LKB1^S431A/S431A^ (S431A/S431A) mice and the *in vitro* kinase activity towards the LKBtide peptide was measured. Immunoprecipitates were also immunoblotted (IB). Assays were performed in duplicate from tissues derived from four mice per genotype and results are means±S.E.M. The broken line represents the background activity as measured with pre-immune IgG. (**F**) AMPKα1 (liver) and AMPKα2 (muscle; EDL) were immunoprecipitated from wild-type LKB1^+/+^ (+/+) and LKB1^S431A/S431A^ (S431A/S431A) mice and the *in vitro* kinase activity towards the AMARA peptide was measured. Immunoprecipitates were also immunoblotted. Assays were performed in duplicate from tissues derived from four mice per genotype and results are means±S.E.M. The broken line represents the background activity as measured with pre-immune IgG.

Knockin mice in which the Ser^431^ that is phosphorylated by PKA and RSK was changed to an alanine to prevent phosphorylation were created and maintained on an inbred C57BL/6J background (Figure 5A). Homozygous LKB1^S431A/S431A^ mice were born at the expected Mendelian frequency (Figure 5B), were of normal size and appearance, and did not display any overt phenotype at least up to 1 year of age (the oldest animals we have analysed). The LKB1^S431A/S431A^ mice are fertile as crosses of homozygous LKB1^S431A/S431A^ animals resulted in viable offspring.

Immunoblot analysis revealed that the LKB1[S431A] mutant was expressed at similar levels to wild-type LKB1 in all of the tissues studied, including the LKB1_long_ and LKB1_short_ forms in testis as well as in MEFs (Figure 5C). Employing an antibody that recognizes LKB1 phosphorylated at Ser^431^ we observed that treat-ment of LKB1^+/+^ wild-type MEFs with either forskolin to activate PKA or PMA to stimulate RSK, induced, as expected, a marked phosphorylation of LKB1 at Ser^431^ (Figure 5D). In parallel experiments, stimulation of LKB1^S431A/S431A^ MEFs with forskolin or PMA, as predicted, did not result in increased detection of LKB1 with the phospho-Ser^431^ antibody. It should be noted that the commercial phospho-Ser^431^ antibody used was the only one we tested that worked well in recognizing mouse LKB1 and also weakly recognized non-phosphorylated LKB1[S431A] in the immunoblot analysis of the LKB1^S431A/S431A^ MEFs. This weak signal was unchanged following stimulation of LKB1^S431A/S431A^ MEFs with forskolin or PMA and was also lost in LKB1-knockout MEFs (Figure 5D, lower panel). High levels of LKB1 Ser^431^ phosphorylation were detected in the spleen, but not in the liver, muscle or kidney of wild-type mice (Figure 5C).

### AMPK is activated normally in LKB1^S431A/S431A^ tissues and MEFs

To assess how the S431A mutation affected LKB1 kinase activity, we immunoprecipitated endogenous LKB1 from muscle and liver extracts and studied kinase activity using the LKBtide peptide substrate. This revealed that loss of Ser^431^ phosphorylation had no effect on catalytic activity (Figure 5E). We next assessed the level of AMPK activity and T-loop phosphorylation in the liver and muscle of littermate wild-type and LKB1^S431A/S431A^ mice. This revealed that the mutation had no significant effect on the basal activity of AMPK (Figures 5C and 5F).

**Figure 6:**
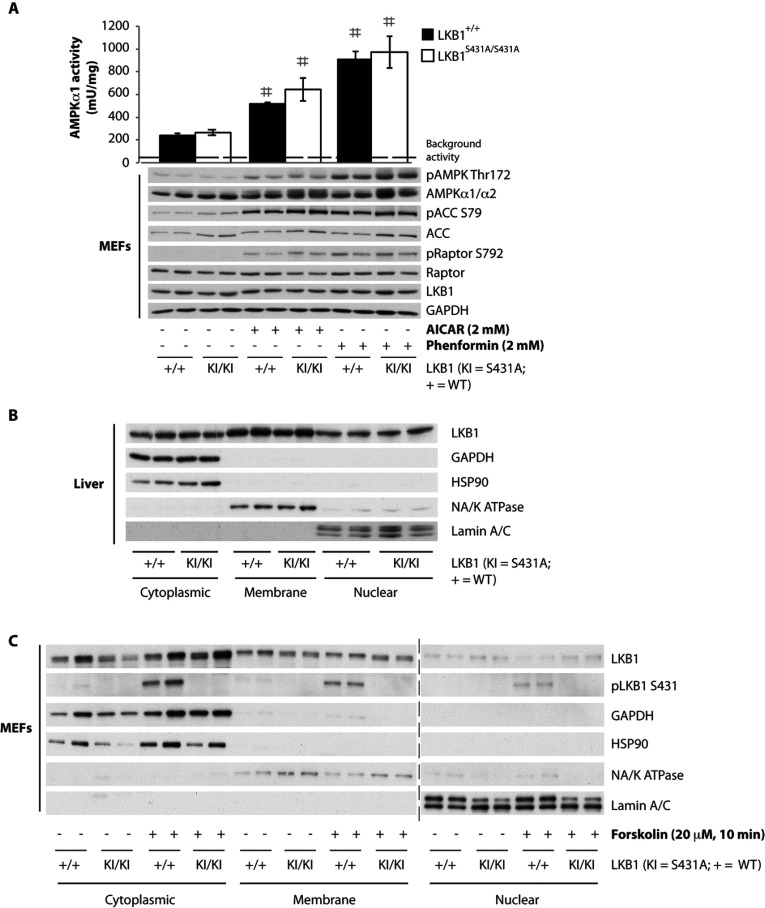
Normal activity of AMPK in MEFs derived from wild-type LKB1^+/+^ (+/+) and LKB1^S431A/S431A^ (KI/KI) mice (**A**) MEFs were treated with 2 mM AICAR or phenformin for 1 h. AMPKα1 was immunoprecipitated from MEF extracts and the *in vitro* kinase activity towards the AMARA peptide was measured. Assays were performed in duplicate for each condition and results are means±S.E.M. The broken line represents the background activity as measured with pre-immune IgG. MEF extracts were immunoblotted with the indicated antibodies. No significant basal phosphorylation was observed for the Ser^431^ site in the LKB1^S431A/S431A^ MEFs (results not shown). #*P*<0.05 treated compared with non-treated cells. Statistical analysis was performed using one-way ANOVA and Tukey's post-hoc test. There was a normal localization of LKB1 in the liver or MEFs derived from wild-type LKB1^+/+^ and LKB1^S431A/S431A^ mice. Livers (**B**) and MEFs (**C**) from wild-type LKB1^+/+^ (+/+) and LKB1^S431A/S431A^ (KI/KI) mice were submitted to subcellular fractionation. Cytoplasmic, membrane and nuclear fractions were immunoblotted with the indicated antibodies. No significant basal phosphorylation was observed for the Ser^431^ site in the LKB1^S431A/S431A^ liver fractions (results not shown). The broken line indicates that the samples were processed in parallel on different gels.

We also stimulated wild-type and LKB1^S431A/S431A^-knockin MEFs with AICAR and phenformin and observed that mutation of Ser^431^ had no effect on the activation of AMPKα1 or AMPK T-loop phosphorylation induced by either of agonist (Figure 6A). We also investigated by subcellular fractionation whether mutation of Ser^431^ affected the localization of LKB1 in the liver (Figure 6B) and MEFs (Figure 6C). This revealed that the mutation of Ser^431^ had no significant effect on the amount of LKB1 present within the cytoplasmic, membrane and nuclear fractions analysed. Moreover, stimulation of MEFs with forskolin to induce phosphorylation of Ser^431^ also had no effect on the fraction of LKB1 between the cytoplasmic, membrane and nuclear fractions (Figure 6C).

### AMPK-related kinases are activated normally in LKB1^C433S/C433S^ and LKB1^S431A/S431A^ mice tissues

We also measured the activity of five endogenous AMPK-related kinases (SIK3, NUAK1, MARK4, BRSK1 and BRSK2) after their immunoprecipitation from the liver, muscle and brain extracts obtained from littermate wild-type, LKB1^C433S/C433S^ and LKB1^S431A/S431A^ mice. This revealed that mutation of Ser^431^ or Cys^433^ had no effect on the activity of these AMPK-related protein kinases (Supplementary Figures S5 and S6 at http://www.biochemj.org/bj/458/bj4580041add.htm).

## DISCUSSION

The role of LKB1 farnesylation has intrigued since it was first reported over 13 years ago [25,26]. To date the only data indicating a role for LKB1 farnesylation was immunofluorescence localization and subcellular fractionation studies employing overexpressed LKB1 that suggested that mutation of Cys^433^ to prevent farnesylation inhibited the association of LKB1 with the plasma membrane [20,25,29]. As mentioned in the Results section, we were unable to identify a suitable antibody to localize endogenous mouse LKB1 in immunofluorescence studies that did not bind and therefore signal in LKB1-knockout MEFs. It should be noted that previous studies analysing the localization of endogenous LKB1 were undertaken using immunohistochemistry [32,53] rather than immunofluorescence, which does not offer sufficiently high resolution to distinguish between membrane and cytoplasmic localization [54]. In future work it would be important to identify antibodies that allow the robust detection of endogenous LKB1 by immunofluorescence. Nevertheless, our subcellular fractionation studies indicate that levels of LKB1 associated with the membrane fraction of the liver and MEFs derived from LKB1^C433S/C433S^ mice were significantly reduced compared with wild-type mice (Figure 2G). It should be noted that in these studies the fractionation performed was relatively crude and the cytoplasmic fraction contained endomembranes as judged by immunoblotting for ER, endosome and lysosome markers (Supplementary Figure S2). Therefore our data does not rule out that the farnesylated LKB1 present within the cytoplasmic fraction is actually localized to the endomembranes. Further work is warranted to investigate this. Moreover, the membrane fraction is a high-speed pellet and probably contains other non-plasma membrane components. Consistent with this was also the detected contamination with the ER as indicated by immunoblotting using the GRP78/Bip marker (Supplementary Figure S2). Further work would be required to establish whether LKB1 localized within this fraction was indeed associated with the plasma membrane. Nevertheless, our finding that in the liver and MEFs of LKB1^C433S/C433S^-knockin mice the levels of LKB1 present within the membrane fraction are significantly reduced supports previous data that farnesylation serves to promote the membrane association of LKB1. Our data also reveal that knockin mutation of Ser^431^ to alanine or forskolin-induced phosphorylation of Ser^431^ does not affect LKB1 subcellular fractionation (Figure 6). We have found relatively small amounts of LKB1 present within the nuclear subcellular fractions (Figures 2G, 6B and 6C), which is consistent with previous work that suggests that association with STRAD and MO25 results in nuclear-exclusion promoting cytosolic localization and that the vast majority of LKB1 is believed to be part of this complex [6,35].

Immunoprecipitation studies employing the anti-LKB1 farnesylation-specific antibody indicated that the bulk, if not all, of the LKB1 expressed in tissues/cells studied (muscle, liver, testis and brain and MEFs) was farnesylated. Immunoblotting studies also revealed that LKB1 present in the cytosolic fraction was similarly farnesylated as the LKB1 present within the membrane fractions (Figure 2G). Much previous work has established that farnesylation of proteins serves to facilitate membrane association, but does not operate to permanently anchor proteins at the plasma membrane [27,28]. Thus many farnesylated proteins, such as LKB1, are frequently localized in the cytosol as well as other compartments in addition to being found at the plasma membrane [27,28].

The approach that we employed to raise an antibody against an *in vitro*-farnesylated peptide, could be used in the future to raise other prenylation-specific antibodies to aide with analysing the roles that this post-translational modification plays more generally. Our immunoprecipitation studies indicate that the anti-LKB1 farnesylation-specific antibody generated is not selective for LKB1 and immunoprecipitates at least 13 other proteins terminating with a CAAX motif that are likely to be farnesylated [27,28] as well as eight Rab GTPases that terminate in a XCXC or XXCC consensus sequence in which both cysteine residues become geranylgeranylated [44] (Supplementary Table S2). It is therefore probable that other farnesylation-specific antibodies raised in the future would also recognize subgroups of prenylated proteins. Nevertheless, the anti-LKB1 farnesylation-specific antibody generated in the present study could have a use in studying prenylation of some of the other proteins listed in Supplementary Table S1 that we have found it to recognize. These include DnaJ homologues, isoforms of Rab, Rap, Ras and Rho, the mitotic regulator spindly, and the protein kinase MAPKAPK3 that is activated by p38 MKK (MAPK kinase) [55], which, to our knowledge, has not previously been reported to be farnesylated, but terminates in a CAAX motif.

Strikingly, in all of the tissues/cells examined (liver, muscle and MEFs), ablation of LKB1 farnesylation significantly inhibited both basal and AMPK activity stimulated by treatments such as AICAR (Figure 3A and Supplementary Figure S4), muscle contraction (Figures 4A and 4B) and phenformin (Supplementary Figure S4) that are dependent on LKB1 [10–12,34]. In LKB1^C433S/C433S^ mice hepatocytes, reduced activation of AMPK resulted in a significantly blunted suppression of AICAR-induced lipid synthesis (Figure 3B). The effect on lipid synthesis was most striking at a low doses of AICAR (50 μM) and was not observed at the higher doses tested (Figure 3B). It might be possible that there is a ‘threshold’ for activity required to elicit AMPK/ACC1-mediated lipid synthesis and thus, even though there is still lower AMPK activation in LKB1^C433S/C433S^ mice hepatocytes, high doses of AICAR stimulate AMPK beyond the threshold. This would also probably explain why the profoundly reduced activation of AMPK seen following contraction of muscle taken from LKB1^C433S/C433S^ mice was not sufficient to inhibit glucose uptake (Figures 4C and 4F), as it is known to be very sensitive (i.e. lower ‘threshold’) to AMPK activation, which is thereby triggered by low levels of AMPK activation [34,52]. Previous work has shown that in another mouse model displaying a reduced AMPK activity, in which LKB1 was expressed at only approximately 10% of the normal levels in most tissues, these animals were viable and displayed no overt phenotypes or marked glucose intolerance [34,56]. Consistent with our observations in muscle taken from LKB1^C433S/C433S^ mice, contraction of LKB1 hypomorphic muscle triggered normal glucose uptake despite a significantly reduced activation of AMPK [34]. Complete knock out of LKB1 in the muscle was required to inhibit glucose uptake [34,57,58]. Overall, the moderate reduction in basal, as well as stimulated, AMPK activity probably explains why LKB1^C433S/C433S^ animals are viable and display no overt phenotypes or intolerance to glucose or AICAR (Supplementary Figure S3). We also noticed that the reduced activation of AMPK in hepatocytes from LKB1^C433S/C433S^-knockin mice affected the phosphorylation of raptor, but not ACC (Figure 3A). This is probably due to ACC being a very good substrate for AMPK and therefore only a small degree of AMPK activation is required to trigger maximal phosphorylation of this substrate.

**Figure 7:**
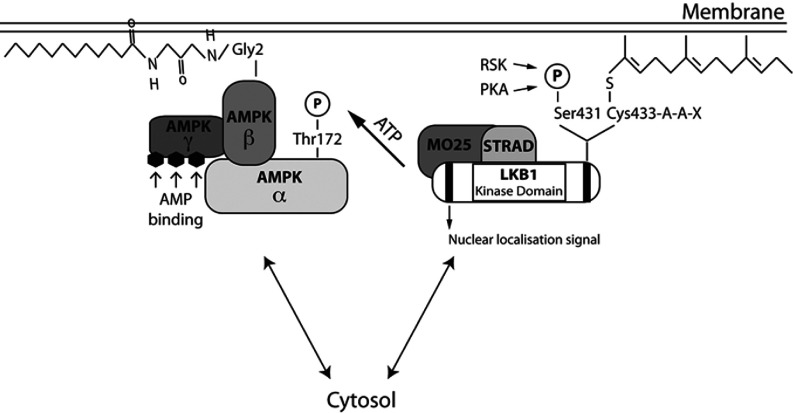
Schematic representation of the potential mechanism by which LKB1 farnesylation and myristoylation of AMPKβ might operate to promote the interaction of LKB1 and AMPK by localizing these enzymes on a two-dimensional membrane surface The LKB1–STRAD–MO25 complex is anchored to the membrane through an LKB1 farnesylation motif. AMPKβ myristoylation promotes the kinase association to the membrane. In the presence of an increased ratio of AMP/ATP or other metabolic signals that deplete the intracellular ATP levels, the co-localization of these two kinases at the membrane allows LKB1 to phosphorylate AMPK on its T-loop residue (Thr^172^) and thereby fully activates AMPK in response to a metabolic signal.

An important question concerns the mechanism by which LKB1 farnesylation promotes the activation of AMPK. In parallel studies we found that endogenous immunoprecipitated mutant LKB1[C433S] phosphorylated the artificial substrate LKBtide peptide and activated the recombinant heterotrimeric AMPK complex to the same extent as wild-type LKB1 (Figures 2E and 2F). This indicates that ablation of LKB1 farnesylation does not affect the intrinsic kinase catalytic activity or ability of LKB1 to interact with and activate the recombinant AMPK complex. So how might membrane association of LKB1 promote activation of AMPK? One possibility is whether the previously reported co-translational N-terminal myristoylation of Gly^2^ in AMPKβ1 [59] and AMPKβ2 [60] could function to co-localize the AMPK complex and farnesylated LKB1 on a two-dimensional membrane surface. Myristoylation of proteins has similarities to farnesylation in that it functions to loosely stabilize the association of proteins at membranes without permanently fixing them at this location [61]. If LKB1 and AMPK were co-localized on a two-dimensional surface of a membrane, this could potentially greatly boost the efficiency at which these enzymes interact compared with a three-dimensional environment (Figure 7). Consistent with the notion that a pool of AMPK is activated at the plasma membrane, Kemp and colleagues have reported that stimuli that trigger activation of AMPK via an LKB1-dependent pathway, such as glucose deprivation, promote the plasma membrane association of AMPK [60]. Moreover, they observed that mutation of the myristoylated Gly^2^ on the AMPKβ subunits inhibited membrane association as well T-loop Thr^172^ phosphorylation and hence the activation of AMPK [60].

Further work is warranted to establish the role that the myristoylation of AMPKβ subunits plays in regulating membrane localization and LKB1 phosphorylation. This might be best addressed by creating knockin mice in which Gly^2^ of AMPKβ1 and AMPKβ2 is mutated to ablate myristoylation. If a pool of AMPK was activated by LKB1 at the plasma membrane, there is some analogy with other signalling systems, such as the PDK1 (phosphoinositide-dependent kinase 1) network. The PDK1 pro-tein kinase possesses a PH (pleckstrin homology) domain that promotes membrane association through its ability to interact with the 3-phosphoinositide second messenger product of the class 1 PI3K (phosphoinositide 3-kinase) pathway PtdIns(3,4,5)*P*_3_ [62]. This helps to localize PDK1 with its Akt substrate that also possess a PtdIns(3,4,5)*P*_3_-binding PH domain at the plasma membrane. Binding of Akt to PtdIns(3,4,5)*P*_3_ induces a marked conformational change that exposes Thr^308^, the residue that PDK1 phosphorylates [62]. *In vitro*-reconstitution studies showed that co-localization of PDK1 and Akt on lipid vesicles containing PtdIns(3,4,5)*P*_3_ markedly increased in the efficiency at which PDK1 can phosphorylate and activate Akt [63,64]. On the basis of *in vitro* studies, a knockin mutation that prevents PDK1 from interacting with PtdIns(3,4,5)*P*_3_ was expected to have a major effect on Akt activation, but instead only reduced Akt activation ~2-fold in the tissues and cell lines investigated [65,66]. This magnitude of effect is analogous with the effect that the loss of farnesylation of LKB1 has on the activation of AMPK observed in the present study. Subsequent work has revealed that the relatively small effect on Akt activation of ablating binding of PDK1 to PtdIns(3,4,5)*P*_3_ is probably due to a second mechanism that brings Akt and PDK1 together and that is mediated by PDK1 possessing a docking site that recognizes Akt after it is phosphorylated at Ser^473^ by mTORC2 (mTOR complex 2) [67]. It is probable that for the activation of master signalling components, such as Akt and AMPK, several alternate mechanisms will operate to bring these enzymes together with their upstream regulators. Thus ablation of any of these mechanisms individually may only have a moderate overall effect. The observation that a large 10-fold reduction in the expression of LKB1 only leads to a relatively small effect on AMPK [34], emphasizes how efficient the activation of AMPK by LKB1 is in cells. Promoting the co-localization of LKB1 and AMPK at the plasma membrane may compromise only one of the several cellular mechanisms that bring these enzymes together.

As for LKB1, a significant proportion of PDK1 is localized in the cytoplasm even following the activation of PI3K pathways, where it plays a critical role in phosphorylating cytosolic substrates, such as p70 S6K (S6 kinase) and RSK isoforms, that are not known to reside within membranes. Evidence suggests that the soluble inositol phosphates, such as inositol(1,3,4,5,6)*P*_5_ and Ins*P*_6_ (inositol hexakisphosphate), which are present at micromolar levels serve to anchor a portion of cellular PDK1 in the cytosol as they interact with the PH domain of PDK1 at nanomolar affinities, thereby preventing PDK1 from interacting with 3-phosphoinositides at the membrane [68]. It would be interesting to see if analogous systems operate to anchor LKB1 in the cytoplasm away from the plasma membrane, for example, by binding to the prenylation motif. Cytoplasmic LKB1 may have a key role in the activation of AMPK-related kinases, which our data suggests is not influenced by LKB1 farnesylation as the activity of AMPK-related kinases we have assayed was not impaired in tissues from LKB1^C433S/C433S^ animals.

Previous work has suggested that LKB1 and its phosphorylation of Ser^431^ controlled axon specification in the developing nervous system in response to BDNF by promoting the activation of the AMPK-related kinases BRSK1/BRSK2 (SAD-B/SAD-A) [37,38]. This work raised a lot of excitement as it suggested that phosphorylation of LKB1 at Ser^431^ was a focal point of a signalling network that linked extracellular determinants of neuronal morphogenesis through a cascade of at least five kinases, TrkB (tropomyosin-related kinase B), PKA, LKB1, SAD-A and SAD-B, to effectors that polarize neurons [37,38]. The finding that LKB1^S431A/S431A^ mice are viable and display no obvious overt phenotype or co-ordination/balance abnormalities does cast doubt on whether phosphorylation of Ser^431^ is critical in controlling the regulation of neuronal polarity. For example, BRSK1/BRSK2 (SAD-B/SAD-A) double-knockout mice that exhibit a major neuronal polarity phenotype die within 2 h of birth, display little spontaneous movement and have a weak response to tactile stimulation [69]. Further work is clearly warranted to study neuronal polarization and axon specification in the brain cells/tissue of LKB1^S431A/S431A^ mice. Our finding that BRSK1/BRSK2 (SAD-B/SAD-A), as well as other AMPK-related kinases assayed (Supplementary Figure S6), as well as AMPK are normally active in the brain and other tissues of LKB1^S431A/S431A^ mice also suggests that phosphorylation of Ser^431^ is not critical for the activation of these enzymes.

The finding that LKB1[S431A] is normally active is also consistent with previous work suggesting that mutation of Ser^431^ has no effect on the catalytic activity or ability of LKB1 to associate with STRAD and MO25 [6,31,34,35]. In *Drosophila* loss-of-function mutations in the *LKB1* gene caused defects in polarity of the oocyte, and this was rescued by low level expression in the germ line of wild-type LKB1, but not by mutation of the residue homologous with Ser^431^ (Ser^535^) [29]. This observation might suggests that there would be problems with development of LKB1^S431A/S431A^ embryos; however, we observe that these mice were born at the expected Mendelian frequency, suggesting that mammalian embryonic development is not significantly affected by ablation of this phosphorylation site (Figure 5B). Interestingly, the residue equivalent to Ser^431^ is conserved in all species where LKB1 has been reported, including *C. elegans* where the CAAX motif is not conserved, suggesting strongly that phosphorylation of this residue must indeed have a significant function. Although we have failed to observe a phenotype in LKB1^S431A/S431A^-knockin mice, it should be stressed that our analysis does not rule out that the LKB1^S431A/S431A^ or even the LKB1^C433S/C433S^ animals do indeed display a phenotype that we have not noticed. It is also possible that significant phenotypes would emerge if the knockin mice were challenged with conditions that we have not investigated.

Much previous work has also focused on the loss of LKB1 in tumours. Pathological examination of organs from LKB1^S431A/S431A^ or LKB1^C433S/C433S^ mice of up to 1 year of age revealed no detectable spontaneous tumour formation, suggesting that neither of these mutations alone is sufficient to inhibit the LKB1 tumour-suppressor function and lead to spontaneous tumour development. This conclusion is consistent with the data from Fogarty and Hardie [36] that showed that overexpression in HeLa cells (that lack LKB1 expression) of LKB1[S431A] together with STRAD and MO25 inhibited the cell cycle to the same extent as wild-type LKB1. In future work it would be of interest to cross LKB1^S431A/S431A^ or LKB1^C433S/C433S^ mice with tumour-prone mice to investigate whether these mutations have any effect on the ability of LKB1 to operate as a tumour suppressor.

## Online data

Supplementary data
