# Supplementary material for: Investigation of LKB1 Ser431 phosphorylation and Cys433 farnesylation using mouse knockin analysis reveals an unexpected role of prenylation in regulating AMPK activity
Source: Biochem J. 2014 Jan 20;458(Pt 1):41–56. doi: 10.1042/BJ20131324 (PMC3898322; doi:10.1042/BJ20131324)
Supplement: Supplementary data [file bj4580041add.pdf]

Biochem. J. (2014) **458**, 41–56 (Printed in Great Britain) doi:10.1042/BJ20131324

online data

SUPPLEMENTARY ONLINE DATA

Investigation of LKB1 Ser[431] phosphorylation and Cys[433] farnesylation using mouse knockin analysis reveals an unexpected role of prenylation in regulating AMPK activity

Vanessa P. HOUDE*[1], Maria Stella RITORTO*, Robert GOURLAY*, Joby VARGHESE*, Paul DAVIES*, Natalia SHPIRO*, Kei SAKAMOTO*[2] and Dario R. ALESSI*[1]

*MRC Protein Phosphorylation and Ubiquitylation Unit, College of Life Sciences, University of Dundee, Dow Street, Dundee DD1 5EH, U.K.

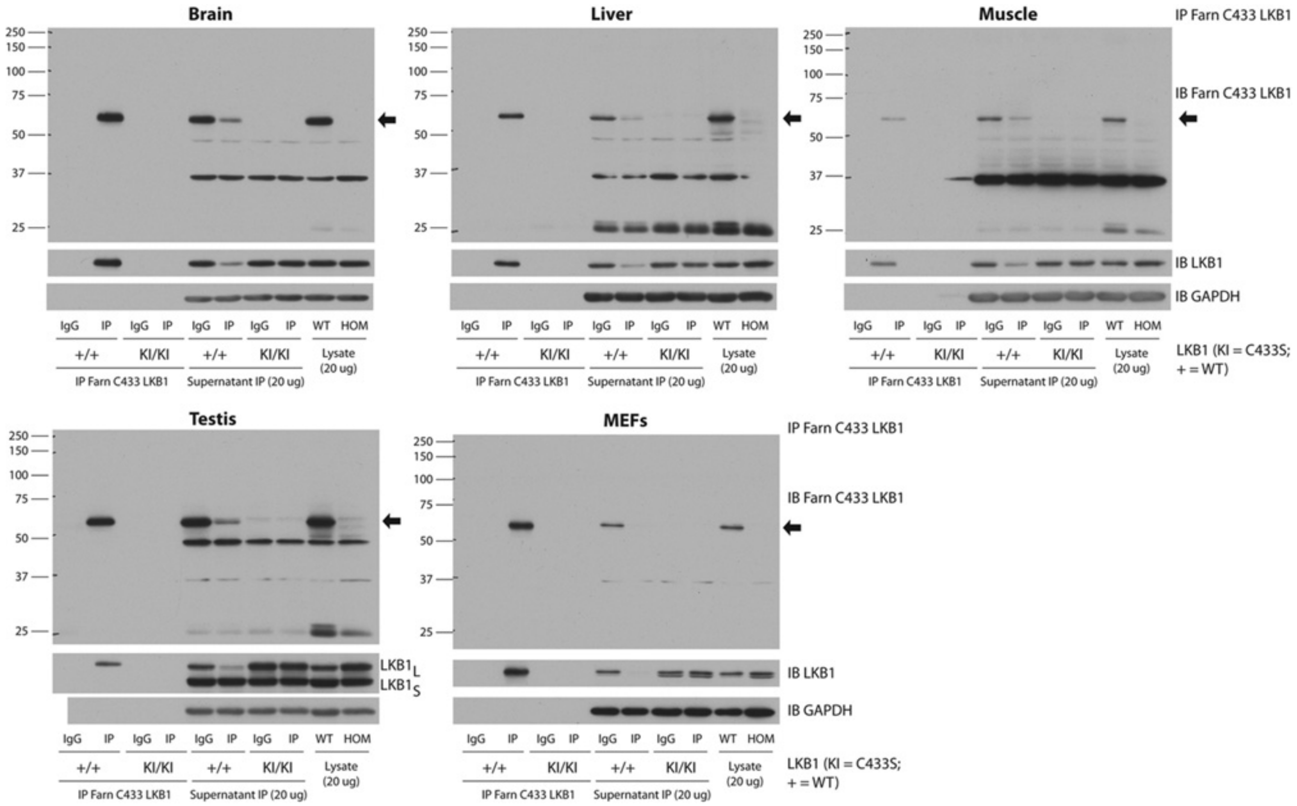

Figure S1 Immunoprecipitation studies using the anti-LKB1 farnesylation-specific antibody from the muscle, liver, brain and testis as well as MEFs taken from wild-type LKB1$^{+/+}$ (+/+) and LKB1$^{C433S/C433S}$ (KI/KI) mice

Immunoprecipitates (IP), as well as immunoprecipitate supernatants and total lysates, were subjected to immunoblotting using anti-LKB1 and anti-LKB1 farnesylation-specific (LKB1 Farn C433) antibodies. The arrows indicate farnesylated C433 LKB1. Molecular mass is given on the left-hand side in kDa. HOM, homozygous LKB1$^{C431/C431}$; WT, wild-type.

[1] Correspondence may be addressed to either of these authors (email v.houde@dundee.ac.uk or d.r.alessi@dundee.ac.uk).
[2] Current address: Nestlé Institute of Health Sciences SA, Campus EPFL Quartier de l'innovation, bâtiment G 1015 Lausanne, Switzerland.

© 2014 The Author(s)
The author(s) has paid for this article to be freely available under the terms of the Creative Commons Attribution Licence (CC-BY) (http://creativecommons.org/licenses/by/3.0/) which permits unrestricted use, distribution and reproduction in any medium, provided the original work is properly cited.

Biochemical Journal www.biochemj.org

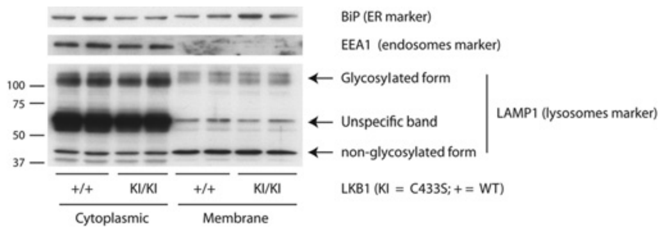

Figure S2 Liver cytoplasmic and membrane fractions from wild-type LKB1$^{+/+}$ (+/+) and LKB1$^{C433S/C433S}$ (KI/KI) mice were immunoblotted with the indicated antibodies to determine the presence of endomembranes (endosomes, lysosomes and ER) in the cytoplasmic fraction preparation

Molecular mass is given on the left-hand side in kDa.

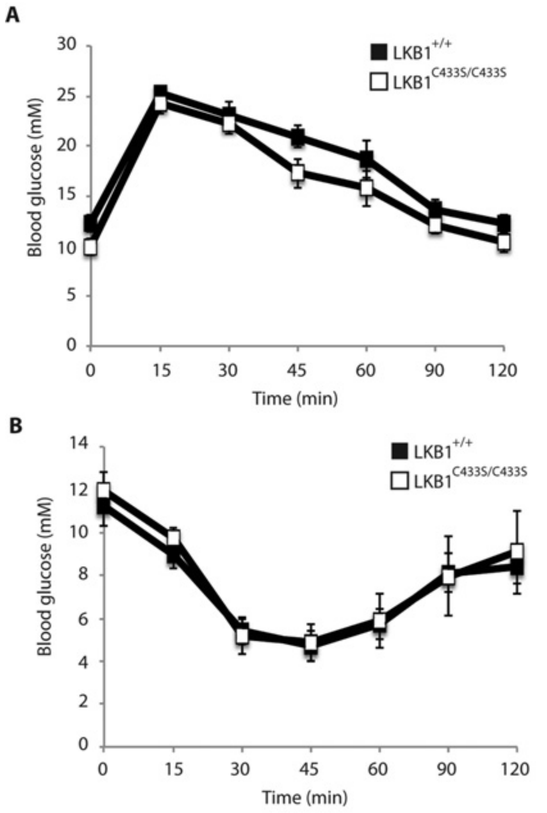

Figure S3 Glucose metabolism in 2-month-old LKB1$^{C433S/C433S}$ mice

(**A**) Glucose tolerance test. Blood glucose concentration was measured from 2-month-old male wild-type LKB1$^{+/+}$ and LKB1$^{C433S/C433S}$ knockin mice fasted for 6 h and injected intraperitoneally with glucose (2 g/kg of body mass). Results are the means ± S.E.M. ($n = 4$). (**B**) AICAR tolerance test. Blood glucose concentration was measured from 2-month-old male wild-type LKB1$^{+/+}$ and LKB1$^{C433S/C433S}$ knockin mice fasted for 6 h and injected intraperitoneally with AICAR (250 mg/kg of body mass). Results are the means ± S.E.M. ($n = 4$).

© 2014 The Author(s)
The author(s) has paid for this article to be freely available under the terms of the Creative Commons Attribution Licence (CC-BY) (http://creativecommons.org/licenses/by/3.0/) which permits unrestricted use, distribution and reproduction in any medium, provided the original work is properly cited.

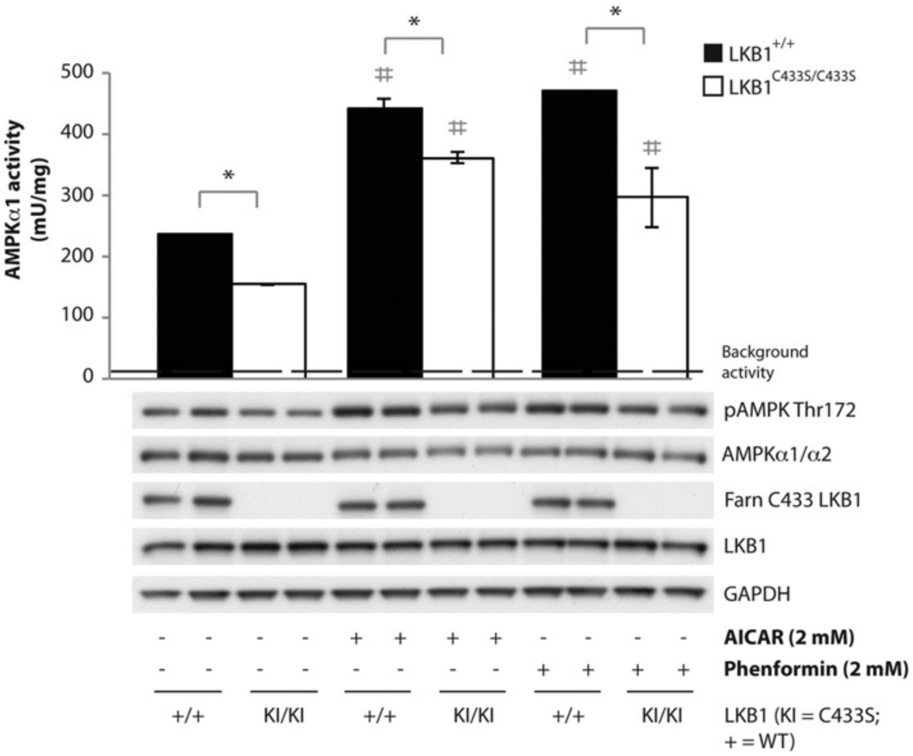

Figure S4 Impaired activity of AMPK in MEFs derived from wild-type LKB1$^{+/+}$ (+/+) and LKB1$^{C433S/C433S}$ (KI/KI) mice

MEFs were treated with 2 mM AICAR or phenformin for 1 h. AMPKα1 was immunoprecipitated from MEF extracts and the *in vitro* kinase activity towards the AMARA peptide was measured. Assays were performed in duplicate for each condition and results are means $\pm$ S.E.M. The broken line represents the background activity as measured with pre-immune IgG. MEF extracts were immunoblotted with the indicated antibodies. *$P < 0.05$ LKB1$^{+/+}$ compared with LKB1$^{C433S/C433S}$ mice within each condition. #$P < 0.05$ treated compared with non-treated cells. Statistical analysis was performed using one-way ANOVA and Tukey's post-hoc test. LKB1 Farn C433, anti-LKB1 farnesylation-specific antibody.

© 2014 The Author(s)
The author(s) has paid for this article to be freely available under the terms of the Creative Commons Attribution Licence (CC-BY) (http://creativecommons.org/licenses/by/3.0/) which permits unrestricted use, distribution and reproduction in any medium, provided the original work is properly cited.

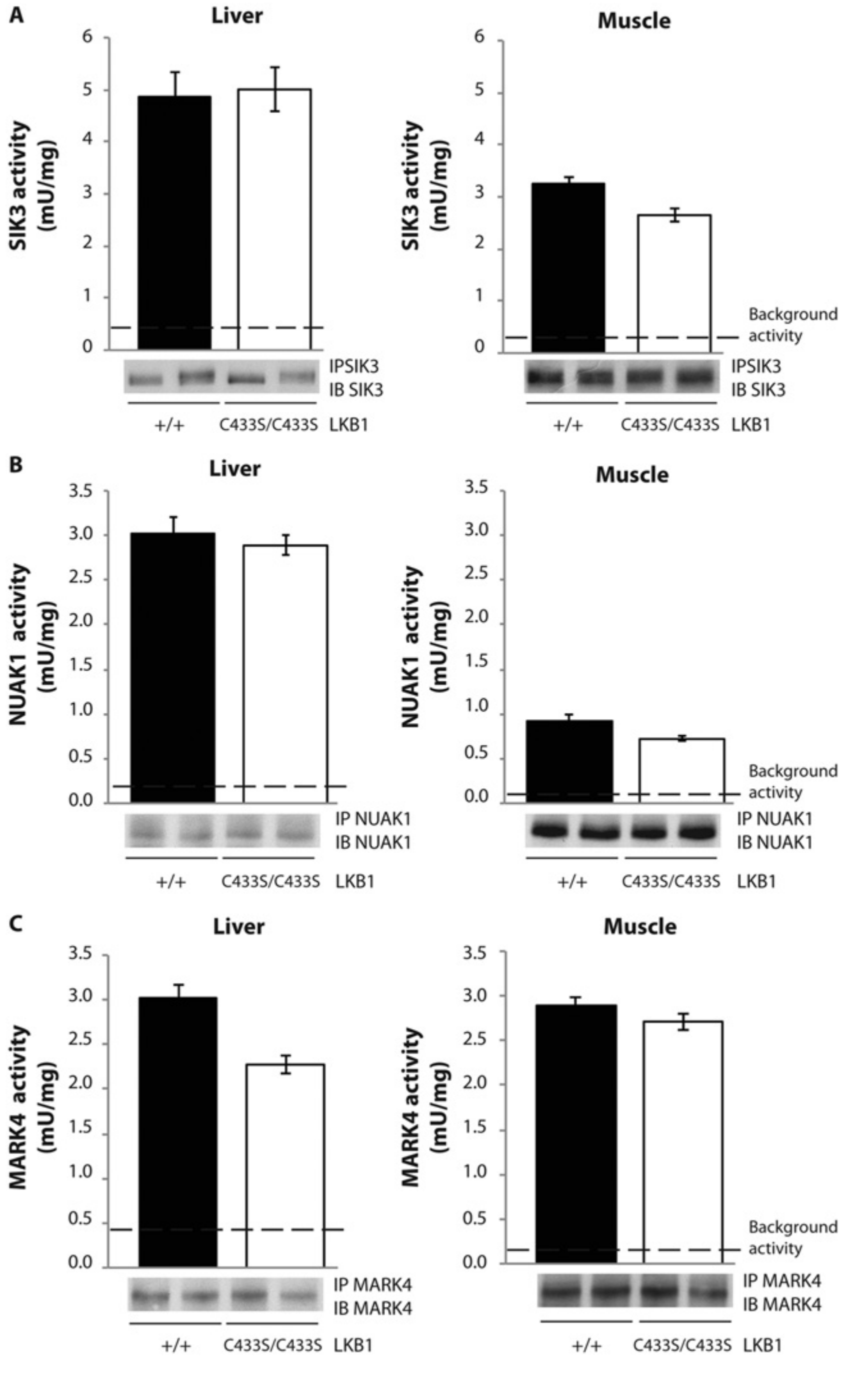

Figure S5 Activity of AMPK-related kinases in mouse tissues taken from wild-type LKB1$^{+/+}$ and LKB1$^{C433S/C433S}$ mice

(**A**) SIK3 was immunoprecipitated (IP) from liver and muscle (EDL) from wild-type LKB1$^{+/+}$ (+/+) and LKB1$^{C433S/C433S}$ (C433S/C433S) mice and the *in vitro* kinase activity towards the Sakamototide peptide was measured. Immunoprecipitates were also immunoblotted (IB). Assays were performed in duplicate from tissues taken from six mice per genotype and results are means ± S.E.M. (**B**) as in (**A**), except that NUAK1 was immunoprecipitated. (**C**) As in (**A**), except that MARK4 was immunoprecipitated. The broken line represents the background activity as measured with pre-immune IgG. All *P* values for the differences between LKB1$^{+/+}$ compared with LKB1$^{C433S/C433S}$ mice were not significant.

© 2014 The Author(s)
The author(s) has paid for this article to be freely available under the terms of the Creative Commons Attribution Licence (CC-BY) (http://creativecommons.org/licenses/by/3.0/) which permits unrestricted use, distribution and reproduction in any medium, provided the original work is properly cited.

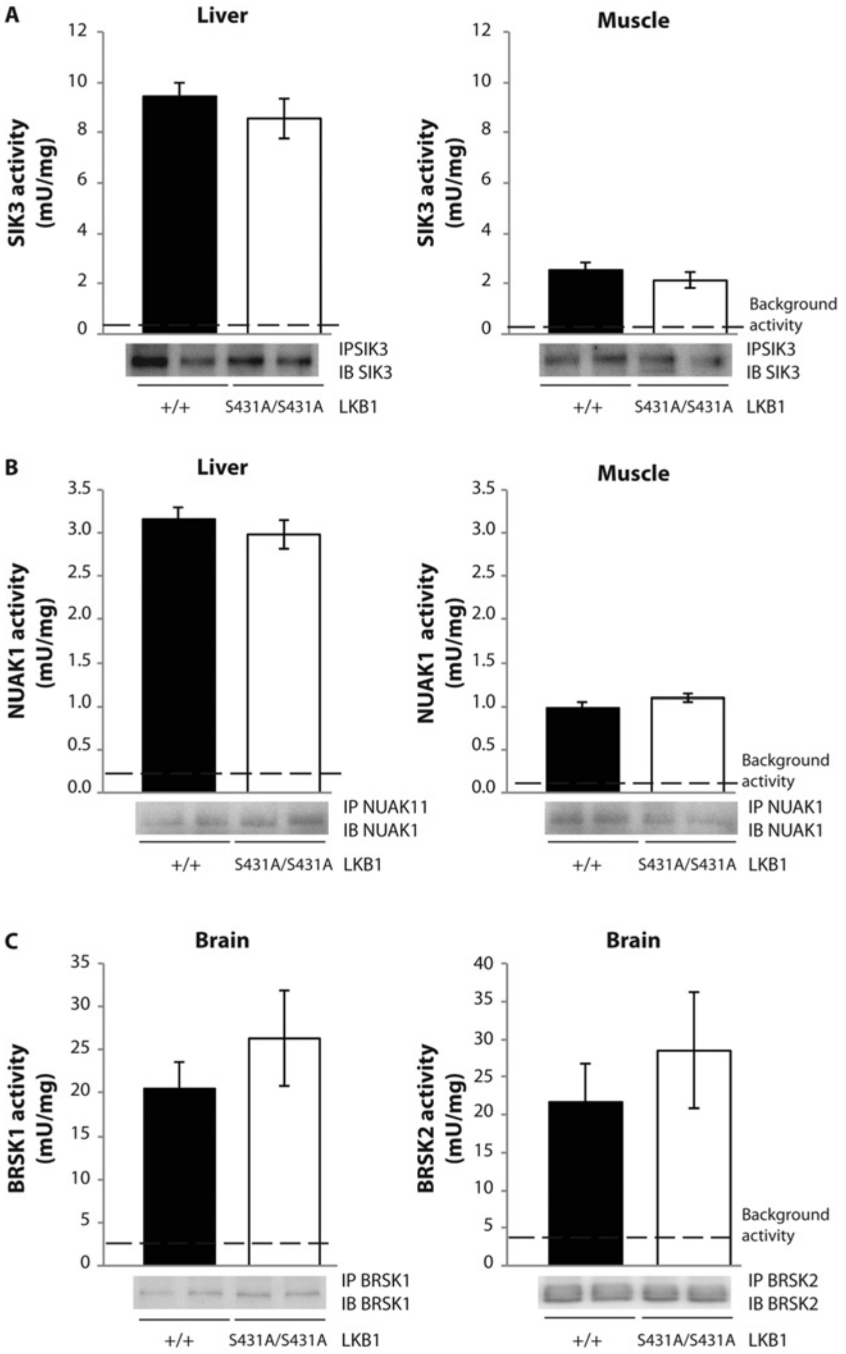

Figure S6 Activity of AMPK-related kinases in mouse tissues taken from wild-type LKB1$^{+/+}$ and LKB1$^{S431A/S431A}$ mice

(**A**) SIK3 was immunoprecipitated (IP) from liver and muscle (EDL) from wild-type LKB1$^{+/+}$ (+/+) and LKB1$^{S431A/S431A}$ (S431A/S431A) mice and the *in vitro* kinase activity towards the Sakamototide peptide was measured. Immunoprecipitates were also immunoblotted (IB). Assays were performed in duplicate from tissues derived from four mice per genotype and results are means ± S.E.M. (**B**) as in (**A**), except that NUAK1 was immunoprecipitated. (**C**) As in (**A**), except that BRSK1/BRSK2 was immunoprecipitated. The broken line represents the background activity as measured with pre-immune IgG. All *P* values for the differences between LKB1$^{+/+}$ compared with LKB1$^{C433S/C433S}$ mice were not significant.

© 2014 The Author(s)
The author(s) has paid for this article to be freely available under the terms of the Creative Commons Attribution Licence (CC-BY) (http://creativecommons.org/licenses/by/3.0/) which permits unrestricted use, distribution and reproduction in any medium, provided the original work is properly cited.

Table S1 Endogenous LKB1 was immunoprecipitated with the anti-LKB1 farnesylation-specific antibody in samples of mice liver, muscle, brain and testis and MEFs derived from wild-type and C433S/C433S knockin mice and analysed by MS to identify potential binding/interacting proteins

Results are representative data from two independent experiments. The proteins listed below are known to be prenylated (farnesylated or geranylgeranylated) except for MO25, STRAD and MAPKAPK3.

(a) Liver

	Liver LKB1$^{+/+}$		Liver LKB1$^{C433S/C433S}$		
Protein name	Mascot score	Number of peptides	Mascot score	Number of peptides	SwissProt accession number
LKB1	1012	64			Q9WTK7
MO25	1216	91			Q06138
STRADα	287	21			Q3UUJ4
STRADβ	899	49			Q8K4T3
DnaJ homologue subfamily A member 2	1238	100	878	73	Q9QYJ0
DnaJ homologue subfamily A member 1	2016	136	1658	124	P63037
Ras-related protein Rap2A	758	33	531	33	Q80ZJ1
Rab14	226	19	244	20	Q91V41
Rab35	198	10	192	9	Q6PHN9

(b) Muscle

	Muscle LKB1$^{+/+}$		Muscle LKB1$^{C433S/C433S}$		
Protein name	Mascot score	Number of peptides	Mascot score	Number of peptides	SwissProt accession number
LKB1	698	54			Q9WTK7
MO25	891	86			Q06138
STRADα	113	14			Q3UUJ4
STRADβ	569	37			Q8K4T3
DnaJ homologue subfamily A member 2	1857	138	457	55	Q9QYJ0
MAPKAPK3	787	66	104	17	Q3UMW7

(c) Brain

	Brain LKB1$^{+/+}$		Brain LKB1$^{C433S/C433S}$		
Protein name	Mascot score	Number of peptides	Mascot score	Number of peptides	SwissProt accession number
LKB1	3228	184			Q9WTK7
MO25	3166	173			Q06138
STRADα	2013	152			Q3UUJ4
STRADβ	689	30			Q8K4T3
DnaJ homologue subfamily A member 2	1885	122			Q9QYJ0
DnaJ homologue subfamily A member 1	1144	78			P63037
Ras-related protein Rap2A	758	33	531	33	Q80ZJ1
Rap2C	431	18	289	21	Q8BU31
Rheb	350	24	266	25	Q921J2
HRAS	195	10	77	6	Q61411
Rab8A	164	10	176	7	P55258
Rab6B	233	16	193	15	P61294
Rab10	228	13	199	8	P61027
NRAS	148	10	199	16	P08556
Rho-related GTP-binding protein RhoG	52	2	82	6	P84096

(d) Testis

	Testis LKB1$^{+/+}$		Testis LKB1$^{C433S/C433S}$		
Protein name	Mascot score	Number of peptides	Mascot score	Number of peptides	SwissProt accession number
LKB1	1574	103			Q9WTK7
MO25	2588	131			Q06138
STRADα	1614	120			Q3UUJ4
STRADβ	246	20			Q8K4T3
DnaJ homologue subfamily A member 2	2597	161	2630	229	Q9QYJ0
DnaJ homologue subfamily A member 1	2280	122	2578	176	P63037
Rab33B	87	3	70	2	O35963
Rab39A	87	2	70	2	Q8BHD0
Rab15	87	2	73	4	Q8K386
Rab18	173	12	105	7	P35293
Rab35	134	7	73	4	Q6PHN9
Rab1b	100	9	73	9	Q9D1G1
Rab8A	99	6	73	6	P55258
MAPKAPK3	56	11	99	14	Q3UMW7

© 2014 The Author(s)
The author(s) has paid for this article to be freely available under the terms of the Creative Commons Attribution Licence (CC-BY) (http://creativecommons.org/licenses/by/3.0/) which permits unrestricted use, distribution and reproduction in any medium, provided the original work is properly cited.

Table S1 Continued

(e) MEFs

Protein name	MEF LKB1$^{+/+}$ Mascot score	MEF LKB1$^{+/+}$ Number of peptides	MEF LKB1$^{C433S/C433S}$ Mascot score	MEF LKB1$^{C433S/C433S}$ Number of peptides	SwissProt accession number
LKB1	1384	64			Q9WTK7
MO25	1328	76			Q06138
STRADα	1094	62			Q3UUJ4
STRADβ	48	5			Q8K4T3
DnaJ homologue subfamily A member 2	1591	118	1170	70	Q9QYJ0
DnaJ homologue subfamily A member 1	1039	54	727	40	P63037
Spindly	536	40	447	32	Q923A2
MAPKAPK3	149	12	330	23	Q3UMW7
Rho-related GTP-binding protein RhoC	570	26	416	16	Q62159
Rab18	143	3	231	8	P35293
Rab35	120	3	101	5	Q6PHN9

Table S2 Sequence alignment of proteins with the CAAX motif that have been immunoprecipitated with the anti-LKB1 farnesylation-specific antibody from tissues or MEFs derived from wild-type and C433S/C433S knockin mice

The C-terminal cysteine (C) is the residue that is prenylated, A is any aliphatic amino acid and X determines if the protein is farnesylated (X = M, S, Q, A or C) or geranylgeranylated (X = L or E).

Protein name	Protein motif	Alignment of the last six amino acids
LKB1	CAAX	SACKQQ
DnaJ homologue subfamily A member 2	CAAX	VQCAHQ
DnaJ homologue subfamily A member 1	CAAX	VQCQTS
Ras-related protein Rap2A	CAAX	SACNIQ
HRAS	CAAX	CKCVLS
NRAS	CAAX	SPCVLM
Rheb	CAAX	SSCSVM
Rap2C	CAAX	TTCVVQ
Rho-related GTP-binding protein RhoG	CAAX	RSCILL
Rho-related GTP-binding protein RhoC	CAAX	RGCPIL
Rab18	CAAX	GYCSVL
Rab8A	CAAX	FRCSLL
MAPKAPK3	CAAX	QGCNNQ
Spindly	CAAX	TQCSQQ
Rab35	XCC	RKKRCC
Rab10	XCC	WKSKCC
Rab1B	XCC	ASGGCC
Rab14	CXC	REGCGC
Rab6B	CXC	EGGCSC
Rab15	CXC	SKTCWC
Rab33B	CXC	AVTCWC
Rab39A	CXC	RKECFC

Received 4 October 2013/14 November 2013; accepted 3 December 2013
Published as BJ Immediate Publication 3 December 2013, doi:10.1042/BJ20131324

© 2014 The Author(s)
The author(s) has paid for this article to be freely available under the terms of the Creative Commons Attribution Licence (CC-BY) (http://creativecommons.org/licenses/by/3.0/) which permits unrestricted use, distribution and reproduction in any medium, provided the original work is properly cited.
